# Access to Orphan Drugs: A Comprehensive Review of Legislations, Regulations and Policies in 35 Countries

**DOI:** 10.1371/journal.pone.0140002

**Published:** 2015-10-09

**Authors:** Todd Gammie, Christine Y. Lu, Zaheer Ud-Din Babar

**Affiliations:** 1 University of Auckland, Auckland, New Zealand; 2 Department of Population Medicine, Harvard Medical School and Harvard Pilgrim Health Care Institute, Boston, Massachusetts, United States of America; Mario Negri Institute for Pharmacology Research, ITALY

## Abstract

**Objective:**

To review existing regulations and policies utilised by countries to enable patient access to orphan drugs.

**Methods:**

A review of the literature (1998 to 2014) was performed to identify relevant, peer-reviewed articles. Using content analysis, we synthesised regulations and policies for access to orphan drugs by type and by country.

**Results:**

Fifty seven articles and 35 countries were included in this review. Six broad categories of regulation and policy instruments were identified: national orphan drug policies, orphan drug designation, marketing authorization, incentives, marketing exclusivity, and pricing and reimbursement. The availability of orphan drugs depends on individual country’s legislation and regulations including national orphan drug policies, orphan drug designation, marketing authorization, marketing exclusivity and incentives such as tax credits to ensure research, development and marketing. The majority of countries (27/35) had in place orphan drug legislation. Access to orphan drugs depends on individual country’s pricing and reimbursement policies, which varied widely between countries. High prices and insufficient evidence often limit orphan drugs from meeting the traditional health technology assessment criteria, especially cost-effectiveness, which may influence access.

**Conclusions:**

Overall many countries have implemented a combination of legislations, regulations and policies for orphan drugs in the last two decades. While these may enable the availability and access to orphan drugs, there are critical differences between countries in terms of range and types of legislations, regulations and policies implemented. Importantly, China and India, two of the largest countries by population size, both lack national legislation for orphan medicines and rare diseases, which could have substantial negative impacts on their patient populations with rare diseases.

## Introduction

Orphan drugs are medicines or vaccines intended to treat, prevent or diagnose a rare disease. Examples of rare diseases include genetic diseases, rare cancers, infectious tropic diseases and degenerative diseases. The definition of rare diseases varies across jurisdictions but typically considers disease prevalence, severity and existence of alternative therapeutic options. In the United States (US) rare diseases are defined as a disease or a condition which affects fewer than 200,000 patients in the country (that is, 6.4 in 10,000 people) [1] while the European Union (EU) identifies a rare disease as a life-threatening or chronically debilitating condition affecting no more than 5 in 10,000 people [1]. 6000–8000 rare diseases are estimated to exist today, affecting approximately 6–8% of the world’s population [1–4]. A recent systematic review [5] of cost-of-illness studies on 10 rare diseases (including cystic fibrosis and haemophilia) found overall limited information published [5]. The availability of information ranges from none to little between diseases and the estimated total cost of illness also ranges substantially between studies conducted in different countries, for example, lifetime costs of cystic fibrosis in Germany was estimated at €858,604 per patient in 2007, while US data suggest €1,907,384 in 2006 [5].

Availability and access to medicines are important to reduce morbidity and mortality of rare diseases. For instance, until the recent availability of pirfenidone, a lung transplant was the only treatment option for patients with idiopathic pulmonary fibrosis, a rare disease with a 50% chance of survival at 3 years [6]. Despite the need and importance of availability and access to orphan drugs, there is a paucity of available treatments for rare diseases. Less than one in ten patients with rare diseases receives disease-specific treatment [7]. Drug development for rare diseases is often limited by the prohibitive cost of investing in an original pharmaceutical agent with poor profit potential given the small patient size per rare disease indication. Under human rights principles, patients with rare diseases have equal rights to medicines as other patients with more prevalent disease (e.g., diabetes). They should not be excluded from gaining benefits from medical advances just because of the rarity of their illness [1, 3]. In this context, many governments and authorities have established legislations, regulations and policies to encourage the research and development of orphan drugs [3, 4, 8] and to address licensing regulations and pricing and reimbursement of these drugs [4, 8–10]; such economic and regulatory incentives are important public health decisions.

It is important to understand regulatory and policy initiatives for orphan drugs that exist in countries and their differences to improve research and policy development for treatment of rare diseases. However, existing articles in this field predominantly either summarized regulations and policies in a single country or continent, or discussed the effect of a single or few regulations/policies influencing access to these important medicines. The aim of this study was to review, as comprehensively and systematically as possible, the range and types of existing legislations, regulations and policies that are utilised by countries to enable the availability and accessibility of orphan drugs.

## Methods

### Search Strategy

The PRISMA guidelines for conducting systematic reviews were followed [11]–(S1 Appendix: PRISMA Checklist). The literature search was undertaken between November 01, 2014 and January 15, 2015 to identify published peer-reviewed articles in English. The databases searched (by TG) included: Medline (1998–2014), PubMed (1998–2014), Google Scholar (1998–2014), Springer Links (1998–2014), Scopus (1998–2014) and the Cochrane Library (1998–2014). We also searched the following journals: Health Policy (1998–2014), Pharmaeconomics (1998–2014), Orphan Drugs: Research (1998–2014) and the Orphanet Journal of Rare Diseases (1998–2014). A search strategy was developed and implemented under the leadership of ZB and CYL. Keywords included the following: (“Access” or “Availability” or “Accessibility”) and (“Orphan” or “High Cost”) and (“Orphan Medicines” or “Orphan Drugs” or “Orphan Pharmaceuticals”) and (“Drugs” or “Medicines” or “Pharmaceuticals”) and (“Regulation” or “Policy”). The keywords were combined and integrated in database and journal searches. Search results (‘hits’) by database and journal are detailed in (S2 Appendix: Search Results). Within the conducted search “Boolean Operator” rules were utilised. The terms used were searched using ‘AND’ to combine the keywords listed and using ‘OR’ to remove search duplication where possible. References of retrieved articles were assessed for relevant articles that our searches may have missed.

### Article Selection and Data Collection

**Fig 1:**
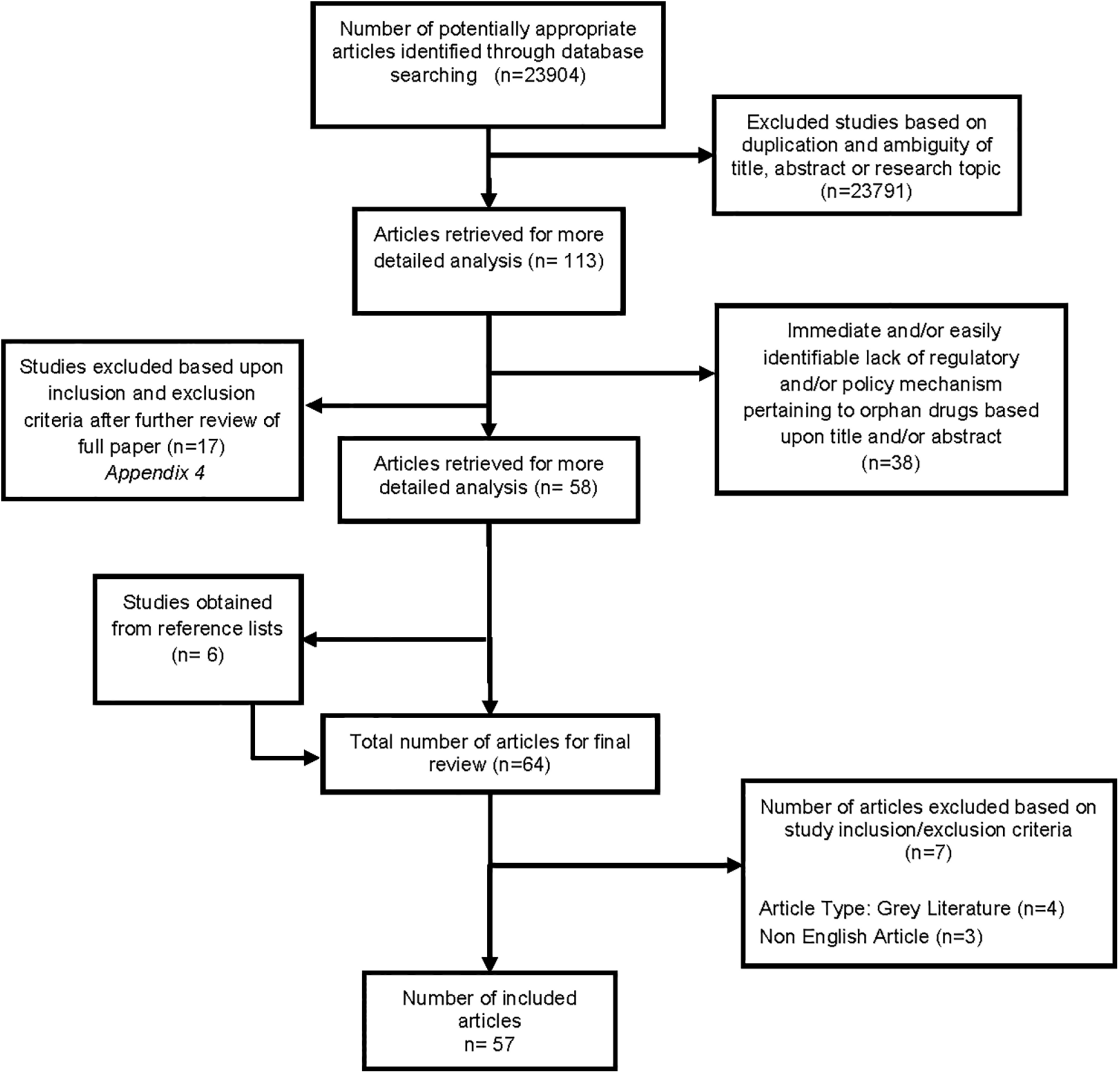
Diagrammatic Representation of Study Selection Flow conducted according to the PRISMA (Preferred reporting items for systematic reviews and meta-analyses) Statement.

From the database/journal searches 23904 titles/abstracts were retrieved. The title and abstract of all retrieved articles were reviewed by lead author (TG) for relevance. Subsets of research results were checked by a second author (ZB or CYL). If there was any ambiguity with regards to the paper, the full-text article was retrieved and reviewed for relevance. After removing duplicates and titles/abstracts unrelated to orphan drugs or rare diseases, we identified 113 peer-reviewed, English-language articles. We included original articles, reviews, commentaries and opinions if they described legislations/regulations/policies for orphan drugs and relevant health services. Of these, only 58 articles were relevant to legislations/regulations/policies for orphan drugs; thus these articles were read in full by TG, with guidance from ZB and CYL. Six more articles were identified from references of the retrieved articles; thus 64 articles were considered against our study inclusion and exclusion criteria with no significant bias found that would affect the cumulative evidence reported (Table 1). Based on these criteria, a further 7 articles were excluded and 57 articles were included for final analysis (Fig 1).

**Table 1:** Study inclusion & exclusion criteria.

*No*.	*Category*	*Inclusion Criteria*
1	Year of release	1998–2014
2	Publication Type	Full text articles in peer-reviewed scientific journals and in English.
3	Countries Covered	Countries with a publicly funded health system and ability to institute policy and regulation in various methods to facilitate access to orphan medicines. Orphan drug policy and regulation implemented by countries which exist outside of these domains which facilitate access to orphan medicines will also be considered in the review.
4	Kinds of Medicines	Orphan medicines, drugs for rare diseases, medicines developed to diagnose, prevent or treat rare diseases.
5	Definition and issues to include	Orphan medicines, rare diseases, specialised clinicians, biologicals, (patient) access, policy & regulation. Definitions of rare diseases and orphan medicines,(Patient) access, drug availability or accessibility, Marketing authorization, approval, Legislation, policy, regulation, Licensing, pricing, health technology assessment, reimbursement, Research, development, production, marketing
6	Methodology and topic of research	Review of peer reviewed journal articles investigating political and regulatory mechanisms globally by which patients suffering from rare diseases gain access to orphan medicines. Investigating: Policy & Regulation: current range of interventions including marketing authorisation/approval, licensing, pricing, reimbursement, research/development/production incentives, cost effectiveness evaluation and others impacting and facilitating patient access to orphan drugs. Patients: mechanisms of access, impact of cost, health education or exposure of relevant information, supply of medicines regarding orphan medicines & access
7	Outcomes of regulation or policy	Policy or regulatory initiatives, programmes, committees or other established mechanisms that influence and thus facilitate patient access to orphan medicines.
8	Bias	No presence of issues in study design, methods, data collection, analysis or any other factor of the study or article that could lead to bias of the individual study.
No.		*Exclusion Criteria*
1		Articles that are not published in English
2		News Reports
3		Did not describe any specific legislations/regulations/policies for orphan drugs

Data collected on individual articles included: author, objective or aim if any, included countries, article type, dates of data collection or article publication, research methodology if any, collected data if any, and key findings/comments if any. We summarized legislations/regulations/policies for orphan drugs by country. After the extraction of relevant information, a narrative synthesis was undertaken.

### Analysis

We reviewed the literature systematically to ensure that a narrative synthesis produced was sourced from the most complete collection of relevant literature possible. Thematic analysis of the articles was conducted, with the addition of new regulation/policy categories as needed, and relevant sub-categories created for examination until no more themes were identified and saturation was deemed to be reached. Using regulation/policy categories generated by this analysis, we described the range and types of legislation/regulation/policy in each included country that affect the availability and access to orphan drugs.

For the purposes of this review, availability of and access to orphan drugs were each defined as follows. *Availability* of orphan drugs was defined as whether an orphan drug had obtained a relevant marketing authorization (and orphan designation if necessary) [3]. *Access* to orphan drugs was defined as the enabling of individuals in their financial and physical ability to obtain and receive relevant care involving orphan medicines. Access was commonly determined by coverage status, reimbursement, and price [3].

## Results

The included 57 articles involved 35 countries (21 EU countries and 5 Asian countries); these included the US, Canada, Europe, Australia, Taiwan, Singapore and Japan. We did not find any reporting legislations/regulations/policies for orphan drugs in Latin America and African countries. We noted that there had been few original articles (22 of 57) in this field. Our review of the 57 articles generated 6 themes with 13 subcategories. Themes included national orphan drug policy, orphan drug designation, orphan drug marketing authorization, marketing exclusivity, incentives, pricing and reimbursement. These themes describe the political or regulatory mechanism utilised and the relevant influence with regard to patient access to orphan medicines. Table 2 summarises these categories. We summarized the environment for availability of and access to orphan drugs in each of the included countries by these categories (Table 3). (S3 Appendix: General Characteristics of Included Studies) summarized articles included in this study. We summarized our findings by theme below.

**Table 2:** Themes and subthemes emerged from the 57 articles included in the study.

Themes	Sub-Categories
National Orphan Drug Policy	Legislation, National Rare Disease Plans, Cross-border Regulation, Orphan Drug Designation
Orphan Drug Marketing Authorization	Accelerated Procedures
Incentives	Financial Incentives, Non-Financial Incentives
Marketing Exclusivity	Monopolisation
Pricing	Free vs Fixed Pricing
Reimbursement	Health Technology Assessment, Co-Payments, Post marketing surveillance, Managed Entry Agreements

**Table 3:** Legislations, Regulations and Policies for Orphan Drugs by Country.

Country Covered (Reference)	Orphan Drug Legislation	National Plan for Orphan Drugs/Rare Diseases	Orphan Drug Designation	Independent Orphan Drug Market Authorization	Market Exclusivity	Financial Incentives (Country specific)	Non-Financial Incentives (Country Specific)	Pricing	Reimbursement 1. Procedures and/or coverage 2. HTA Criteria
Australia [3, 12–14]	Yes	No	Yes	Yes	No	Yes—Fee Reduction for Marketing Authorization Approval	Pre-licensing access, Regulatory Assistance	Fixed	1. Reimbursement under Australia’s life-saving drug programme, 2. Cost-effectiveness (Under consideration of Australia’s Life-Saving Drug Programme)
Austria [10, 15]	Yes (EU)	No	Yes (EU)	Yes (EU)	EU– 10 Years	No	Free scientific advice, free protocol assistance	Reference Pricing	1. Physicians entitled to prescribe medicines in the Austrian Reimbursement Code–Orphan Drugs often require prior approval 2. Cost-effectiveness
Belgium [10, 16–18]	Yes (EU)	Yes	Yes (EU)	Yes (EU)	EU-10 Years	Tax exemptions	No	Price negotiations	1. Reimbursement drug decisions Ministry of Social Affairs and committee of doctors for orphan medicinal products 2. Therapeutic advantage, Budget Impact, importance in clinical practice
Bulgaria [19–22]	Yes (EU)	Yes	Yes (EU)	Yes (EU)	EU-10 years	No	Pre-licensing access	Reference Pricing	1. Orphan drugs reimbursed by the MoH or NHIF. 2. Cost-effectiveness
Canada [1, 3, 13]	No	No	No	No (Accelerated Review is Possible)	No	Yes (tax incentives, fee reductions for marketing authorization)	Pre-licensing access scientific advice- protocol assistance and/or development consultation, regulatory assistance	Reference pricing (regional negotiation)	1. 80% to 100% reimbursement provided by the Public Service Health Care Plan, with 100% after a drug’s costs after the patient has reached a co-payment threshold of $Can3000 in a calendar year for approved orphan drugs. 2. Cost effectiveness, safety, therapeutic advantage
China [23]	No	No	No	No	No	No	Yes- Ability to conduct smaller clinical trials or waive the trial if necessary	Free	1. Self-funded by patients ‘out of pocket’ with assistance from NGO’s or patient foundations. Reimbursement determined by the National Reimbursement Drug List–Set by the NDRC, CFDA, Ministry of Human Resources & Social Security & the Ministry of Finance 2. Cost-effectiveness
Czech Republic [10, 21]	Yes (EU)	Yes	Yes (EU)	Yes (EU)	EU- 10 Years	No	None	Fixed (Maximum reimbursed price set at 70–99% of the public price including taxes (PPIT))	1. 100% coverage for positively reimbursed orphan drugs. Also includes a special reimbursement regime. Regulated by Health Insurance and a Medical Professional 2. Cost effectiveness
Denmark [10, 24]	Yes (EU)	No	Yes (EU)	Yes (EU)	EU- 10 Years	No	Scientific advice, free protocol assistance, pre-licensing access	Free	1. 100% of approved orphan drugs at hospitals, needs based co-payments for pharmacy prescription 2. Cost- effectiveness
Estonia [10, 25]	Yes (EU)	Yes	Yes (EU)	Yes (EU)	EU– 10 Years	No	No	Free (No policy)	1. 50–100% reimbursement by the Estonia Health Insurance Fund 2. Therapeutic Advantage
Finland [10, 25]	Yes (EU)	No	Yes (EU)	Yes (EU)	EU– 10 Years	No	Free administrative/ scientific (protocol) advice	Free–With justification	1. Basic reimbursement of 35%, with 65%–100% for certain diseases or conditions. 2. Cost-effectiveness
France [3, 4, 16]	Yes (EU)	Yes	Yes (EU)	Yes (Intra-national and EU)	EU– 10 Years	Tax exemptions	Pre-licensing access, scientific advice, free protocol assistance	Price negotiations	1. 65% to 100% for reimbursed orphan drugs–Complementary health insurance often completes reimbursement 2. Therapeutic advantage, unmet need, socio-economic benefits
Germany [3, 4, 26]	Yes (EU)	Yes	Yes (EU)	Yes (EU)	EU– 10 Years	No	Pre-licensing access	Free–Under criteria	1. Automatically reimbursed, based upon a cost benefit analysis by IQWiG (if successful) if no therapeutic alternative–Co-payment of €10 per drug, limited to an annual threshold of 2% of individual yearly net income. 2. Cost-effectiveness
Greece [20, 27]	Yes (EU)	Yes	Yes (EU)	Yes (EU)	EU– 10 Years	No	Pre-licensing access (compassionate use)	Reference pricing	1. Reimbursement by the public insurance system for orphan drugs on the reimbursement list–Patient must pay 50% of cost in excess of the reference price. 2. Cost effectiveness
Hungary [3, 10, 21]	Yes (EU)	No	Yes (EU)	Yes (EU)	EU– 10 Years	No	Pre-licensing access	Free (No policy)	1. Reimbursement under legal special equity procedure 2. Specialised HTA for orphan drugs
India [28]	No	No	No	No	No	No	No	Fixed	1. Predominately self-funded (for orphan medicines)–Patient foundations, NGO’s utilised for funding assistance 2. Cost-effectiveness, clinical efficacy
Ireland [10]	Yes	No	Yes (EU)	Yes (EU)	EU– 10 Years	No	No	Fixed–Decisions made by the corporate pharmaceutical unit in the Health Service Executive	1. Differential—Community and national high tech drug schemes 2. Cost-effectiveness
Israel [13]	No (Orphan drug Government offices established)	No	No	No	No	No	Pre-licensing access (compassionate, off-label use)	Free (No policy)	1. Reimbursement for drugs in the Israeli ‘basket of services’ 2. Cost-effectiveness, Social, Ethical, Legal Implications
Italy [1, 4, 10, 16, 26, 29, 30]	Yes (EU)	Yes (draft)	Yes (EU)	Yes (EU)	EU -10 Years	No	Pre-licensing access, scientific advice, free protocol assistance	Price negotiations (Reference Pricing)	1. Reimbursement (licensed orphan drugs), through a standard pricing/reimbursement process, Law 658 and 5% AIFA special fund) 2. Cost-effectiveness, budget impact, need, existing therapies
Japan [1, 12, 23, 29, 31, 32]	Yes	No	Yes	Yes	10 Years	YesFinancial subsides, tax credits, corporate tax reductions, user fee waivers	Priority review, fast track approval, free protocol assistance	Fixed–Cost plus 10%	1. 100% (30% from insurance companies, 70% from national/ regional governments) for approved orphan drugs 2. Cost effectiveness
Latvia [10, 33, 34]	Yes (EU)	Yes	Yes (EU)	Yes (EU)	EU- 10 years	No	Scientific advice, free protocol assistance)	Free (No policy)	1. 100% for orphan drugs on the reimbursement list or individual reimbursement for up to €14,229. 2. Cost effectiveness, Therapeutic advantage
Macedonia [27]	No	Yes	No	No	No	Fee reduction	Shorter registration period	Reference Pricing	1. Reimbursement by public funds based on HTA (Macedonian or reference based) 2. Cost-effectiveness
Poland [3, 21, 26]	Yes (EU)	Yes	Yes (EU)	Yes (EU)	EU– 10 Years	No	Pre-licensing access	Reference Pricing–With review by the Minister of Health	1. Reimbursement of 100% if successful, conducted with HTA and comprehensive data, price data in other EU countries, also reimbursed through therapeutic programmes. 2. Cost-effectiveness, Therapeutic advantage
Portugal [24]	Yes (EU)	Yes	Yes (EU)	Yes (EU)	EU- 10 Years	No	No	Free (No Policy)	1. NHS reimbursement scheme–All citizens covered for positive drug decisions (on national reimbursement list) 2. Cost-effectiveness
Romania [20, 21, 27]	Yes (EU)	Yes	Yes (EU)	Yes (EU)	EU- 10 Years	No	No	Free (no policy)	1. National Programme for Rare Diseases provides reimbursement for orphan drugs on application. 2. Cost-effectiveness
Serbia [22]	No	Yes	No	No (Accelerated Review/Access is Possible)	No	No	No	Reference Pricing	1. Positive reimbursement list dictates drug payments–by the patient or public funds. 2. Cost effectiveness, Therapeutic advantage
Singapore [1, 35]	Yes	No	Yes, but, Doctor or Dentist designates orphan diseases	Yes (Legislation enables the importation of orphan drugs for a specific rare disease, top registration priority)	10 Years	No	No	Free–Orphan drug legislation has yet to be ‘activated’	1. Reimbursement decisions made by the Centre for Drug Administration (CDA)–Aims to simplify and streamline evaluation of pharmaceuticals in Singapore 2. Cost-effectiveness
Slovakia [3, 10, 21]	Yes (EU)	No	Yes (EU)	Yes (EU)	EU– 10 Years	No	Pre-licensing access	Free (No policy)	1. All authorized orphan drugs reimbursed with a €0.16 co-payment per package 2. Cost effectiveness, Therapeutic advantage
Spain [4, 26]	Yes(EU)	Yes	Yes(EU)	Yes (EU)	EU- 10 years	Reduced rebates	Pre-licensing access	Fixed (Cost plus system)	1. 100% if reimbursement status is approved 2. Therapeutic advantage
Sweden [4, 10, 16, 36]	Yes (EU)	Yes	Yes (EU)	Yes (EU)	EU -10 Years	No	No	Free	1. Reimbursement conducted by public social insurance. If the total cost exceeds 4300 SEK the patient will receive the medicines free of charge 2. Cost-effectiveness, Human value, Solidarity
Switzerland [3]	No	No	Yes	No	No	Tax exemptions	Pre-licensing access (off-label, compassionate use)	Free (No policy)	1. Public reimbursement after a deductible and 10% co-payment (annual co-payment threshold of $646 USD). 2. Cost-effectiveness, Human value, Solidarity
The Netherlands [3, 4, 15, 16, 18, 37]	Yes (EU)	**Y**es	Yes (EU)	Yes (EU)	EU– 10 years	Registration fee waivers	Pre-licensing access	Price negotiations	1.100% reimbursement for approved orphan drugs. The Dutch Policy Rule for Expensive Hospital and Orphan Drugs supports hospitals financially for prescribing orphan drugs. 2. Cost effectiveness–Dispensation from submitting evidence regarding orphan drugs (lack of evidence)
Taiwan [1, 35]	Yes	No	Yes	Yes	10 Years + 2 Years	Grants, Fee Reductions + Others determined by the central competent authority	Regulatory Assistance	Price Negotiations	1. 70% to 100% (for low income families) reimbursement for orphan drugs for rare diseases classified under the Rare Disease Prevention and Medicine Law by the Department of Health/ Bureau of National Health Insurance 2. Cost-effectiveness, clinical efficacy. If approved by US FDA, no clinical trials are required
Turkey [38]	No	No	No	No	No	No	Pre-licensing access	Reference pricing	1. Reimbursement for all orphan drugs successful in entering the market, regardless of licensing. 2. Clinical Efficacy–Orphan Drugs exempt from pharma-economic analysis
United Kingdom [4, 10, 16, 30, 39]	Yes (EU)	Yes	Yes (EU)	Yes (EU)	EU- 10 Years	No	Ongoing debate on pre-licensing access	Fixed–With approval by the Department of Health and rate of return limits imposed by the Pharmaceutical Price Regulation Scheme (PPRS)	1. Reimbursement for approved orphan drugs if incremental cost-effectiveness (ICER) criterion is met. The National Cancer Drug Fund from the National Health Services funds some orphan drugs in the United Kingdom. 2. Incremental cost-effectiveness ratio (ICER) (based on a threshold varying between £20,000 and £30,000 per quality adjusted life year (QALY)–Can be higher for orphan drugs.
United States [1, 3, 23, 31, 40, 41]	Yes	No	Yes	Yes–Fast Track / Priority Review, Accelerated Approval, ‘Breakthrough’	7 Years	Yes (50% tax credits, FDA fee waivers, grants programme)	Scientific advice, protocol assistance, pre-licensing access	Free	1. 95% under Medicare–approved health plans, subject to prior authorization for reimbursement and after total “out of pocket” costs have exceeded $4350 USD. 2. Cost effectiveness–No systematic HTA conducted by US payers for Orphan Drugs

HTA, Health Technology Assessment; MOH, Ministry of Health; NHIF, National Health Insurance Fund; FDA, Federal Drug Administration; IQWiG, Institute for Quality and Efficiency in Health Care

N.B. Countries included within the EU have access to an overall range of incentives offered in over-arching EU legislation for medicines that have been granted an orphan designation by the European Commission including: fees reduction for protocol assistance, marketing authorization application (and the potential for accelerated applications), inspections, annual fees and products utilising the centralised procedure as well as access to free scientific advice regarding marketing authorization and clinical trials.

Cost-effectiveness HTA criteria in decision making includes the importance of clinical efficacy.

### National Orphan Drug Policy

National Orphan Drug Policy was one of the 6 themes identified from our review and included: orphan drug legislation, national rare disease plans, cross-border regulation, and orphan drug designation.

#### Orphan drug legislation

Orphan drug legislation is used by a number of countries to encourage research, development and marketing of orphan drugs [19, 31, 42]. The US was the first country to establish national orphan drug legislation with the Orphan Drug Act passed in 1983 [10]. Japan was the second country to implement orphan drug legislation in 1993 [1, 40]. Australia was also one the first countries to develop orphan drug legislation; Australia’s Therapeutic Goods Act 1990 was amended in 1997 with the full Australian orphan drug policy [3]. EU legislation (Regulation (CE) N°141/2000) for orphan drugs was implemented in 2000. Taiwan and Singapore also include specific legislation pertaining to orphan drugs (Medicines Act Chapter 176, Section 9 & The Rare Disease and Orphan Drug Act respectively) [1]. Orphan drug legislation intends to address the challenges of prohibitive costs of product development and limited profit potential due to the smaller market size for each rare disease [3, 31, 42]. Such legislation includes a variety of incentives to encourage orphan drug research, development and marketing. These often include tax credits for research costs of orphan drugs, several years of marketing exclusivity [1, 12, 23, 29, 31, 40, 42] that prevents marketing approval of a generic drug or brand name for the same rare disease indication, free scientific advice such as protocol assistance, fast track/ priority review for marketing authorization of orphan designated products and pre-licensing access initiatives, including off-label and compassionate use programmes [4, 10, 13, 14, 20].

It is important to note that patient advocacy was instrumental in the formation of orphan drug legislation such as the US Orphan Drug Act and EU regulation (CE) N°141/2000 [12]. Patients frequently form patient organisations as “surrogate pressure groups” and influence prescribers, regulatory agencies and political bodies in matters of availability of and access to orphan drugs [9, 12, 21, 23, 32, 43–45]. For instance, international organisations such as the National Organisation for Rare Disorders (NORD) in the US and the European Organisation for Rare Diseases (EURODIS). These groups focus on improving care of rare diseases through better access to information as well as individual patient access to orphan drugs and associated treatment [14, 21, 22, 27, 32, 43–46]. Patient advocacy groups often lobby third-party payers or governments funding healthcare, to provide full reimbursement of orphan drugs, regardless of their high price [44]. Patient advocacy groups may form partnerships with regulatory agencies, for example, EURODIS with the European Medicines Agency (EMA) [23].

#### National Rare Disease Plans

National plans for rare diseases have a general purpose to create a regulatory framework for access to services, treatment, and information, research stimulation, and patient advocacy [9, 14, 21, 27, 43, 46]. National rare disease plans differ from orphan drug legislation in that they often do not put specific legislation into place. Most often, they indicate the initial 'readiness' of the country to respond in the field of orphan drugs and rare diseases [14, 43]. These plans include a framework and documentation of a national shared vision in the field of orphan drugs [14, 20–22, 27, 46]. For example, five neighbouring European countries, Bulgaria, Greece, Macedonia, Romania and Serbia, have established national plans for rare diseases [27]. The effectiveness of such plans in terms of availability (orphan drug designations & marketing authorizations) and access (low(er) prices & positive reimbursement decisions) may be affected by national purchasing power, budget as well as decision making criteria for pricing and reimbursement policies [10, 20, 22, 27].

#### Cross-border regulation

The EU is unique in that it is the only entity to have a centralised procedure for orphan drug designation and marketing approval extending across its member countries. Cross-border regulation is of particular importance in the context of rare diseases because patients often do not receive treatment due to inadequate access to orphan drugs as well as inadequate availability of related specialised clinicians and facilities domestically [2]. The directive 2011/24/EU clarifies patients’ rights on cross-border healthcare. This directive enables patients with a rare disease within the EU the right to EU wide healthcare services if the national healthcare system is not able to provide the essential treatment domestically within a reasonable timeframe [2]. However, due to differences in national pricing and reimbursement policies across the EU patients still experience differential access to orphan drugs [2].

#### Orphan Drug Designation

Orphan designations are often based upon: severity (life-threatening or chronically debilitating conditions) and unmet need (no therapeutic alternative or the new product provides significant clinical benefit) [4, 47]. This basis is often further split between prevalence or economic criteria [29]. Prevalence criteria consider specific definitions of orphan diseases and individual national patient prevalence, while economic criteria consider whether expected sales of a drug product would cover the initial investment costs associated with research and development [4, 31]. Differences in prevalence criteria are usually the primary reason for differing definitions of rare diseases and orphan drugs across jurisdictions [4]. There is often a lack of quantity and quality of clinical evidence for orphan drugs, due to a limited number of patients for clinical trials [4, 47, 48]. Orphan drug designation may allow drugs for non-orphan diseases to gain market access [14]. Oncology products account for the greatest number of orphan drug designations in the US (32.5% of all orphan designations); similarly in, Europe, Japan and Australia [1, 3, 4, 31].

### Orphan Drug Marketing Authorization

Assessment for marketing authorization of orphan drugs has been critical in promoting the availability of orphan medicines and is often the same as those for non-orphan medicinal products in non-EU countries [4, 12, 16, 28, 39, 41]. For example, in countries such as Australia, the US and Japan, marketing authorization procedures are largely identical to non-orphan drugs [4, 12, 16, 28, 39, 41]. Similarly, procedures are the same for orphan and non-orphan drugs in countries currently without orphan drug legislation in place such as Canada and Israel [1, 3, 13]. However, marketing authorization procedures differ in the EU. In the EU, decisions on orphan designation are made by the Committee for Orphan Medicinal Products (COMP) of the European Medical Agency while marketing authorization decisions are made by the Committee for Medicinal Products for Human Use (CHMP), the same committee for non-orphan drugs. A single marketing authorization is granted by the CHMP, with the aim to ensure patients with rare diseases have equal access to orphan drugs independent of member state. In a study of 11 countries (Australia, Canada, England, France, Germany, Hungary, Netherlands, Poland, Slovakia, Switzerland and the US) by Blankart et al. [3], all implement similar standards for the approval of orphan drugs. In smaller nations such as Serbia or Macedonia, the process is simplified if the drug has been authorized in other larger nations [16, 27, 28, 39]. This may impact timely availability of orphan drugs in smaller nations because pharmaceutical companies tend to apply for authorization in the US or EU first [3]. Countries often rely upon the same studies to assess clinical effectiveness of orphan drugs. Orphan drugs are often evaluated using the same criteria, including severity and unmet need. However, differences are often found in the interpretation of study results, which may affect the outcome of marketing authorizations [3]. “Success rate”, the proportion of orphan medicines that receive marketing approval after receiving an orphan designation was reported to be as low as averaging 10.9% of all orphan designations granted in the EU in the first ten years of EU orphan drug legislation (2000–2010). Success rate proportions were similar in the US, with a 15.9% success rate in the 28 years since implementation of the 1983 Orphan Drug Act [25]. Low success rates are likely due to the differences in approval criteria for orphan drug designation versus those for marketing authorization. Research and development incentives for orphan drugs likely result in large numbers of applications and orphan drug designations. However, currently stricter criteria for marketing authorization mean many products that received an orphan designation may not ultimately be approved for marketing [25].

#### Accelerated Procedures

Some countries have accelerated procedures to ensure timely availability of orphan drugs to the market [3, 4]. These procedures include: priority review, fast-track approval and accelerated approval [29]. Although the process is applicable to both orphan and non-orphan medicines, the process is more accepted for orphan drugs, nevertheless orphan drugs are not automatically qualified for accelerated procedures. In some countries, less rigid criteria are used for the evaluation of the therapeutic value of orphan drugs [48]. Criteria regarding unmet need, severity, and high clinical efficacy must be met for accelerated review. For instance, in the US priority review is granted to orphan drugs that demonstrate major advances in treatment or meet significant unmet need. For instance, iloprost, an orphan drug for treatment of pulmonary arterial hypertension experienced priority review in the US in December 2004, with a positive outcome within 6 months as compared to the regular 10 month assessment period [3]. An accelerated assessment usually takes about half the time needed for the standard marketing authorization process (~150 days versus a year or longer) [3, 29].

### Incentives

There are financial and non-financial incentives to ensure availability and access to orphan drugs. We summarize these below.

#### Financial Incentives

Financial incentives utilised worldwide include: research grants, tax credits/corporate tax reductions, marketing exclusivity, and user fee waivers [1, 12, 17, 31, 41, 42, 49]. These provisions exist as a means to allow firms to recover research and development costs, which would not be possible with sales of orphan drugs given the small market sizes. These incentives generally help to increase the availability of orphan drugs [3]; Blankart et al. [3] found that only 10% of clinical trials for orphan drugs would have been conducted without such financial incentives.

#### Non-Financial Incentives

Non-financial incentives we identified include: fast track approval, pre-licensing access (in the form of compassionate or off-label access) and scientific advice, that is, free protocol assistance and/or development consultation [1, 3, 4, 10, 29]. Garau et al. [4] investigated a selection of seven EU member states and found four countries (France, Italy, Spain and the Netherlands) allow pre-licensing access to orphan drugs but encourage the collection of additional clinical data to prove therapeutic benefit. Pre-licensing allows importation of orphan drugs available in other countries but currently unauthorized in the country. Pre-licensing access is often the most common method for patients accessing orphan drugs in many countries, often through procedures such as ‘named patient procedures’. Such use may be granted to an individual or a group of patients with a serious or life-threatening disease where there is no alternative therapeutic option [4, 29]. The need is determined by the responsible physician and patient [38]; each application is evaluated in light of relevant evidence and the advice of scientific communities [38]. While pre-licensing access may be granted, access by individual patients is rarely reimbursed by public health insurance [3]; an example is Turkey’s national reimbursement scheme that enables both availability (through importation) and access to orphan drugs when these drugs are unavailable, unauthorized and inaccessible [3, 4, 10, 38]. Free scientific advice including protocol assistance is provided by regulatory authorities to increase the quality of clinical trials and study protocols, and increase the likelihood of successful marketing authorization and subsequent reimbursement applications [4, 20, 29].

### Marketing Exclusivity

Marketing exclusivity is generally implemented as part of a package of incentives to encourage pharmaceutical companies in research and development of orphan drugs. This allows firms several years to recover costs for drug development. During the marketing exclusivity period, regulatory agencies cannot approve a generic drug or brand name drug for the same rare disease indication [3, 31]. However, the same drug can receive approval for a different disease indication and no limits are currently put in place globally on the number of drugs that may be selected for the same rare disease profile [3, 31]. Picavet et al. [29] studied orphan drug policies in Europe and found that the period of marketing exclusivity can be challenged if the orphan drug lacks supply, “is sufficient profitable”, or if another drug is “clinically superior” than the existing orphan drug. These exceptions are mirrored in the US and worldwide. However, to date, the EU and the US have not withdrawn market exclusivity status for any drug, despite increasing profitability [3, 4, 31, 40, 44].

#### Monopolisation

Marketing exclusivity exists as a strong incentive for development of orphan drugs worldwide. However, there are concerns regarding monopolisation and the manufacturers’ high prices for orphan drugs [3]. This is because patients with rare diseases often have a high willingness to pay given the limited therapeutic alternatives and the life-threatening or chronically debilitating nature of many rare diseases. Therefore, third party payers are generally forced to pay the manufacturer’s high price, leading to payment of a monopoly based price scheme [3, 4, 29, 31]. Monopoly based pricing schemes can impact patient access to orphan drugs as well as non-orphan drugs given the pressure to contain increasing health (including pharmaceutical) expenditures [3, 4, 19, 20, 23, 27, 29, 31, 33, 36, 44, 50, 51]. However, contrasting evidence regarding the effect of marketing exclusivity on the creation of a market monopoly has been suggested. Turnover of the first orphan drug authorized for a rare disease indication is linked to increased likelihood of ‘follow-on’ orphan drug research and development. Marketing authorization for the first orphan drug may indicate feasible development for future drugs for the same rare disease. Arguments rejecting claims of market monopolies commonly attribute the occurrence of a single orphan drug for a single rare disease on the small market size, with an inability to attract competition [18].

### Pricing

Pricing of orphan drugs is often referred to as ‘black box’ pricing due to the lack of literature on orphan drug pricing mechanisms [25, 30]. Pricing of orphan drugs is unique in that the costs of research and development must be retrieved from a small number of patients. Given this, marketing exclusivity, and the lack of therapeutic alternatives, orphan drugs are relatively expensive, often exceeding €100,000 per patient per year [15, 25, 30, 52] (e.g., Replagal for Fabry Disease, a rare genetic x linked lysomal storage disease, costs on average US$265987.20 per patient per year [3]). Generally, there are no large variations in ex-factory (manufacturer) prices for orphan drugs between countries of different pricing and reimbursement systems [30]. Rather the heterogeneity in price and access to orphan drugs across countries is possibly due to national budget constraints and political pressures. Orphan drugs with multiple orphan indications, those for chronic treatments and those with demonstrated improvements in overall quality of life or survival are associated with higher annual prices. Repurposed orphan drugs, those orally administered and those for which an alternative treatment was available are associated with lower annual prices of treatment [30]. The variability in access and use of orphan medicines is comparable to other newly authorised, non-orphan drugs in the EU [24].

#### Free versus Fixed Pricing

Fixed pricing, adopted by many EU countries and other countries such as Japan and Canada, often involves two methods. The first is reference pricing, whereby a country compares the price requested by the manufacturer with the price in other countries [19]. Countries that use reference pricing tend to have comparable drug prices, [3, 42] but orphan drugs still have relatively high prices. The second example of fixed pricing is prices set at the discretion of governmental and regulatory bodies. These prices remain fixed as the respective agency will ‘fix’ the price at a level it determines optimal. This included measures such as “cost plus” pricing set at the cost of research and development plus a profit percentage [29]. Free pricing sets prices at the manufacturer’s discretion [40], commonly used in the US and Germany [3, 26]. Fixed pricing models tend to exhibit moderate to significantly lower acquisition prices, averaging around 40% less than free pricing models [3, 10].

### Reimbursement

Coverage and reimbursement of orphan drugs have been widely regarded as the most important determinant of patient access to orphan medicines [1, 4, 9, 12, 16, 37, 41, 53, 54]. Orphan drugs that are not covered by insurance systems are practically inaccessible to patients due to their high cost [33, 42], and even when they are covered, patient cost-sharing (through co-payments or coinsurance) can still limit access. This theme includes 4 subthemes: health technology assessment, co-payments, post-marketing surveillance and managed entry agreements.

#### Health Technology Assessment

Health Technology Assessment (HTA) is often utilised to assess the value of medicinal products, including orphan drugs [13, 34, 35, 55, 56]. Criteria most commonly include measures of cost-effectiveness based upon the indices such as quality adjusted life years (QALY) and incremental cost effectiveness ratios (ICERs) [4, 10, 56]. However, standard HTA practices and standards of evidence that require formal cost-effectiveness analyses and randomised controlled trials are often not strictly applied to orphan drugs given the typical lack of data regarding clinical efficacy and the burden of the disease, lack of appropriate diagnosis and trained health professionals, and small patient sizes [3, 4, 29, 54, 57, 58]. Because of these evidence gaps, a higher level of uncertainty on clinical efficacy, safety, incremental cost-effectiveness and budgetary impact is accepted for orphan drugs in many countries [48].

While orphan drugs often do not meet traditional cost-effectiveness criteria, they may be reimbursed by payers in some countries because other factors are taken into account in reimbursement decisions [17]. These include: therapeutic value, budget impact, impact on clinical practice, pricing and reimbursement practices globally, patient organisations, economic importance, ethical arguments and the political climate [17]. Standards of evidence required in reimbursement decisions across countries may explain these differences. One study [4] found that only 69% of 43 potentially available EMA-granted orphan drugs were reimbursed in Sweden. England and Wales saw only 2 positive recommendations by the National Institute for Health and Care Excellence of the 43 available orphan drugs. Of 28 orphan drugs reviewed in Scotland, 15 (54%) were reimbursed. Finally 94% and 100% of all launched orphan drugs were reimbursed in Italy and France respectively. These effects were attributed to differences in pricing and reimbursement strategies as well as decision making criteria by the aforementioned countries [4]. In particular, while France and Italy focus on a standard of evidence that requires proven clinical value and measures of innovation, both countries do not require a formal cost-effectiveness analysis for orphan drugs [4]. These countries consider literature reviews and cohort studies when clinical evidence and cost-effective evidence are limited based on data from manufacturers [4]. While these countries take into account the high price of orphan medicines, they are often still reimbursed due to their relatively low budget impact because of small patient sizes [4]. Countries that require a standard of evidence including a formal clinical and cost-effectiveness analysis often have lower coverage compared to countries that utilise alternative standards of evidence [4].

Additional considerations by countries can also often include “rule of rescue” (value of rescuing a life regardless of cost) and equity of access. “Rule of rescue” and equity of access criteria are often considered in Canada and Israel for orphan drugs [13]. Common to this theme, reimbursement decision-making authorities in Turkey do not require pharmaco-economic analysis for orphan drugs. Furthermore, all orphan drugs in Turkey entering the market are reimbursed without any co-payment [38].

Proposed HTA solutions for orphan drugs include ‘multi-criteria decision analysis’ that considers measures of: rarity, clinical effectiveness, level of research undertaken, level of uncertainty around effectiveness, manufacturing complexity, follow up measures, disease severity, available alternatives and budget impact. Healthcare resources are then allocated on the basis of the performance of the drug against these criteria until the associated budget is consumed [55].

#### Co-Payments

Access to orphan drugs may be affected by considerable patient co-payment or coinsurance [3], which are out-of-pocket costs for patients. Patient co-payments for prescription drugs can be substantial in some countries such as the US, Canada and Switzerland; for instance, monthly co-payment may be as high as $90 for prescription medicines in the US or a coinsurance of ~30% of the drug’s cost. It is important to note that co-payments by patients for these medicines are not found equally among the countries of this review. For example, in countries such as, but not limited to, the Netherlands and Poland, no co-payment is required for drugs, including orphan drugs, included in the national reimbursement list [3]. Countries often have ‘catastrophic coverage’ to protect against the risk of excessive out-of-pocket expenditure. In the US, healthcare plans approved by Medicare cover 95% of drug costs after patient payments of US$4350 per year have been reached [3]. Similarly, in Canada, Public Service Health Care plans see an increase from 80% to 100% of total drug costs after co-payments of US$2814 per patient per year have been reached [3].

#### Post-marketing surveillance

Clinical evidence requirements at the time of orphan designation and marketing approval may be relaxed if post-marketing surveillance programmes are used. These mechanisms are often utilised to enable early approval and access to drugs for a serious or life-threatening illness. These programmes are in place to ensure that if clinical efficacy requirements are not reached, the drug will no longer be provided [48, 57, 59–62]. Sorafenib, an orphan drug for treatment of renal cell carcinoma was as of December 2012 in Italy subject to post-marketing surveillance to ensure the clinical efficacy of the drug in patients following relaxed clinical evidence at time of approval [60].

#### Managed Entry Agreements

Managed entry agreements (MEAs) are being increasingly utilised worldwide as ‘innovative reimbursement approaches’ to fund high cost (orphan) drugs. These require the manufacturer to enter into an agreement with the payer involving negotiations of performance targets based on expectable health improvements. These schemes are utilised as an alternative approach to provide coverage with restrictions for drugs that may not otherwise be covered [60]. MEAs often present in two formats: performance-based schemes and financial-based arrangements [60].

Performance-based schemes aim to provide security of cost-effectiveness and link performance to reimbursement of (orphan) drugs. These schemes can provide reimbursement with an assurance of post-marketing clinical evidence and surveillance [60]. If performance targets are not reached, drug prices are reduced to maintain satisfactory cost-effectiveness relationships [12, 13, 23, 26]. Patient access is enabled under strict criteria. Financial-based schemes exist to address concerns of healthcare payers regarding cost and the budget impact of orphan drugs. Financial-based schemes take a variety of forms including ‘cost capping’ (beyond a cost threshold the drug is provided at a discount or at zero cost), utilisation capping (any number of doses and/or cycles beyond an agreed amount results in financial consequences, eg price volume agreements), and free and/or discounted initiation (treatment is free up to a specified number of doses). One study found that of 7 EU countries, Italy had the highest number of MEAs for orphan drugs, followed by the Netherlands, England and Wales, Sweden, and Belgium; no data were available for France and Germany [60]. The reasons for these differences are unclear but how uncertainty and value are perceived and defined are possible reasons for inter-country differences in the use of MEAs [60]. Orphan drugs including sorafenib (for treatment of renal cell carcinoma), nilotinib (for treatment of gastro intestinal stromal tumours) and temsirolimus (for treatment of renal cell carcinoma) were subject to both performance and financial based MEAs by the Italian Medicines Agency as of December 2012 [4, 60]. The objective of these schemes was to ‘verify and control the appropriateness of the prescription and to circumscribe the level of uncertainty around the drug’ when clinical efficacy was in question [60].

## Discussion

This article reports a comprehensive literature review of legislations, regulations, and policies used in 35 countries to enable the availability of and access to orphan drugs in the last two decades. Existing reviews in this field predominantly either summarized rare disease and orphan drug regulations and policies in a single country or continent, or discussed the effect of a single or few regulations/policies influencing access to these important medicines. The breadth and depth of our review provides important understanding and appraisal of the topic. We summarized the wide range of legislations, regulations, and policies that influence access to orphan drugs on an international scale and examined similarities and differences in legislations/regulations/policies between countries. We identified 6 major types of regulations/policies each with subcategories.

We included 21 countries from the EU. Orphan drug designation, marketing authorization and 10 year marketing exclusivity are common to EU countries. We also noted key differences between the 35 countries included in the review. Differences in pricing and reimbursement policies and budgetary considerations across countries may result in inequities in access to orphan drugs [3, 4, 19, 20, 22, 27].

Overall, the majority of countries (27 of 35) included in this review have in place orphan drug legislation (either independent legislation or legislation as part of the EU) but only 18 countries had established a national plan for rare diseases and orphan drugs. This is likely because a country having established national orphan drug legislation may not require a national plan. It is also possible that national plans are less prevalent because they are at the discretion of the individual country while national legislation for orphan drugs in EU countries is governed by EU regulation (CE) N°141/2000 [12]. Countries without orphan drug legislation are China, India, Canada, Israel, Macedonia, Serbia, Switzerland and Turkey. Notably, China and India–two largest countries each with population in excess of one billion individuals–do not have in place orphan drug legislation and/or a national rare disease plan.

The EU, US, Japan, Taiwan and Australia all have in place independent pathways for marketing authorization of orphan drugs. In these countries orphan drugs tend to meet criteria for accelerated procedures based upon unmet need or disease severity. Accelerated procedures can shorten marketing authorization timeframe almost by half but countries vary in the implementation of accelerated marketing authorization procedures [3, 10, 29]. Other countries have identical processes for marketing authorization of orphan and non-orphan drugs.

Financial and non-financial incentives are commonly used, with only 9 of 35 countries found to have no financial or non-financial incentive of any kind for orphan medicines. A common challenge for many countries is the inability to adequately implement proposed incentives for orphan drugs due to budgetary constraints [20, 22, 27]. Pre-licensing access in the form of compassionate or off-label access to orphan drugs is common (17 of 35 countries) and allows the importation of unauthorized medicines on a named patient or patient group basis. However, when it exists, pre-licensing access is rarely reimbursed by public health insurance (e.g., Turkey) [1, 3, 4, 20, 27, 33, 34, 38, 49, 61] but in such cases, it promotes both the availability of, and access to these medicines regardless of positive marketing authorization or inclusion on a national reimbursement list [4, 38]; thus, there is not always a clear distinction between ‘availability’ and ‘access’ in the field of orphan drugs. Marketing exclusivity of orphan medicines is widely used (26 of 35 countries) and ranges 5 to 10 years on average. Marketing exclusivity is attractive to pharmaceutical companies and policy-makers worldwide in fostering orphan drug research and development. However, the efficacy of marketing exclusivity in promoting patient access orphan medicines is not apparent [3, 42]. The formation of ‘mini-monopolies’ is a major concern [3, 4, 18, 27, 29, 31, 36, 44].

Access to orphan drugs continues to be limited by high prices. Fixed pricing schemes are common across the countries in the review (16 of 35 countries). However, fixed pricing models are not without limitation; price fluctuations may be explained by international purchasing power parity differences [19, 20, 22, 26, 27].

Reimbursement of orphan drugs is probably the most important factor determining patient access to orphan drugs given their high costs [3, 12, 20, 50]. HTA, particularly cost-effectiveness analysis, has important impact on reimbursement decisions for all drugs, including orphan drugs [3, 4, 13, 29, 38, 62]. Twenty-nine countries consider cost-effectiveness in their assessment of orphan drugs (e.g., the United Kingdom) but many also consider other factors such as unmet need, human value and solidarity (e.g., Sweden); thus, countries often accept a “more limited evidence base” for orphan drugs compared to non-orphan drugs. Interestingly, Hungary has established a separate HTA for orphan drugs. There is worldwide debate about the use of HTA and considerations beyond the typical therapeutic benefits, risks, and costs to include a more systematic consideration of ethical/equity factors such as ‘rule of rescue’ [13, 29, 38, 48].

Patient co-payments in some countries such as the US and Canada pose significant barriers to patient access as well as high acquisition costs of orphan drugs [3, 4, 10]. Thus, regardless of orphan drug availability, patient access can often be substantially restricted by out-of-pocket costs [1, 4, 9, 12, 16, 37, 41, 42, 53, 54]. Of the 35 countries in this review, 33 countries provide some reimbursement for orphan drugs; reimbursement is generally dependent upon if orphan drugs are approved in the country or included on the national reimbursement list. In China and India, which have no orphan drug legislation or associated incentives, costs for orphan medicines are largely self-funded by patients ‘out-of-pocket’. Seven of 35 countries (Canada, Germany, Sweden, US, Switzerland, Denmark and Greece) have dedicated co-payments protection programs that provide financial support once an annual amount of co-payment is exceeded to protect against the risk of excessive out-of-pocket expenditure. Interestingly, countries including, but not limited to, Australia, Italy and the Netherlands have special programmes reimbursing orphan drugs (namely, Australia’s Life-saving drug program, Italy’s 5% Agenzia italiana del Farmaco (AIFA) fund for reimbursing orphan drugs not yet marketed, and the Dutch Policy Rule for Expensive Hospital and Orphan Drugs that supports hospitals financially for prescribing orphan drugs).that are separate to their national drug coverage programs for non-orphan drugs [3, 4, 10, 15, 18, 37].

Managed entry agreements are an innovative and increasingly used approach to enable access to high cost orphan and non-orphan drugs in situations where there is a lack of sufficient evidence for coverage of promising technologies that may benefit patients [13, 17, 26, 48, 58–60, 63].

While many developed countries such as the US, Japan, Australia, and EU countries have established a range of legislations/regulations/policies for orphan drugs, many Asian countries fall behind. A few Asian countries such as Japan, Singapore and Taiwan have made developments in this area. In particular, Taiwan reimburses 70% to 100% of the cost of orphan drugs for low income families under the Rare Disease Prevention and Medicine Law by the Department of Health/ Bureau of National Health Insurance [1, 35]. However, importantly, China and India continue to lack national legislation for orphan medicines and rare diseases [1, 12, 23, 28, 29, 31, 35, 40]. Due to their large populations, the lack of developments to support access to orphan drugs has substantial negative impacts on their patient populations with rare diseases [23, 28].

Our study has some limitations. First, publication and outcome reporting bias may have led to the publication or non-publication depending on the nature or direction of the findings [8]. Investigators were limited to the English language literature; publications in other languages were not included. Second, the review study selection included only articles published in peer-reviewed journals; grey literature was excluded. This was to ensure an academic level of accuracy through the peer review process. Finally, while it was beyond the scope of this review to examine the impacts of regulations and policies for orphan drugs, we noted that published evidence was limited and often used measures that were superficial, such as the number of orphan designations, number of orphan drugs granted marketing authorizations, and number of orphan drugs on reimbursement lists. Nevertheless, with consideration of these limitations, the review provides a better understanding of types of legislations, regulations and policies influencing patient access to orphan drugs. Future research on orphan drugs should identify legislations/regulations/policies for orphan drugs from Latin America and African countries. Research is also needed for comparing prices of (a sample of) orphan drugs and numbers of designated vs. marketing approved orphan drugs in light of the differences in legislations/regulations/policies across countries.

## Conclusions

Overall many countries have undertaken a combination of regulations and policies for orphan drugs in the last two decades. While these may enable the availability and access to orphan drugs, there are critical differences between countries in terms of range and types of regulations and policies implemented. The presence of marketing exclusivity remains critical to incentivising research and development of orphan drugs but poses risks, most notably monopolisation and high prices for orphan drugs, which may limit patient access to these needed medicines. Importantly, China and India that each has populations in excess of one billion individuals, lack national legislation for orphan medicines and rare diseases, which could have substantial negative impacts on their patient populations with rare diseases.

## Supporting Information

S1 Appendix PRISMA Checklist. (DOC)

S2 Appendix Search Results. (DOCX)

S3 Appendix General Characteristics of Included Studies. (DOCX)

S4 Appendix Excluded Studies. (DOCX)
