# Supplementary material for: Access to Orphan Drugs: A Comprehensive Review of Legislations, Regulations and Policies in 35 Countries
Source: PLoS One. 2015 Oct 9;10(10):e0140002. doi: 10.1371/journal.pone.0140002 (PMC4599885; doi:10.1371/journal.pone.0140002)
Supplement: S2 Appendix — (DOCX) [file pone.0140002.s002.docx]

**Appendix 2: Search Results**

Number of Search Results (‘Hits’) by Database or Journal

| **DATABASES** | ‘Access’ AND ‘Orphan Drugs or ‘orphan medicines’ OR ‘rare diseases’ AND “Regulation or ‘Policy’ | ‘Orphan drugs’ OR ‘rare diseases’ AND ‘Regulations’ or ‘Policy’ | (‘Access’ or Availability or ‘Accessibility’) AND (‘Orphan’ or ‘High cost’) AND (‘Orphan medicines’ or ‘Orphan drugs’ or ‘Orphan pharmaceuticals’) AND (‘Drugs’ or ‘medicines’ or ‘Pharmaceuticals’) AND (‘Regulations’ or ‘policy’) |
| --- | --- | --- | --- |
| Google Scholar | 17,400 | 17,700 | 7970 |
| Medline | 45 | 235 | - |
| Pubmed | 106 | 1825 | 78 |
| Springer Links | 201 | 468 (288) | 632 |
| Scopus | 83 | 906 | 26 |
| Cochrane Library | - | 2467 | - |
| **JOURNALS** |  |  |  |
| Orphan Drugs: Research | 13 | 13 | - |
| Health Policy | 72 | 72 | - |
| Pharmaeconomics | 30 | 54 | - |
| Orphanet Journal of Rare Diseases | 15 | - | 45 |
