# Supplementary material for: Access to Orphan Drugs: A Comprehensive Review of Legislations, Regulations and Policies in 35 Countries
Source: PLoS One. 2015 Oct 9;10(10):e0140002. doi: 10.1371/journal.pone.0140002 (PMC4599885; doi:10.1371/journal.pone.0140002)
Supplement: S3 Appendix — (DOCX) [file pone.0140002.s003.docx]

**Appendix 3: General Characteristics of Included Studies**

| **Study (First author’s name)** | **Countries Studied, Article Type, Year of Publication** | **Objective (Aim) of Article** | **Research Methods, Collected data** | **Key Findings/Comments** |
| --- | --- | --- | --- | --- |
| Aagaard (2) | European Union, Primary Research, 2014 | To analyse whether directive 2011/24/EU gives patients with rare diseases the right to receive health care services in another member states, reimbursed by the member state of affiliation. | Legal analysis of EU legislation on cross border health care for patients with rare diseases. The study analyses this legal position to identify reimbursement and patient access to orphan drugs in other EU countries based on legal methodologies. | The scope of EU directive 2011/24/EU allows patients access to “safe and high quality cross-border health care services” in theory, however, does not deal with reimbursement timings, lack of necessary marketed orphan drugs or limited access to marketed drugs due to (a lack of) national reimbursement policies across the EU. Public access to these drugs is limited (across borders) due to the majority of these drugs marketed with hospital or specialist physician only restrictions. Access can be restricted in economically weaker countries due to relatively higher prices. |
| Barak (12) | United States, European Union, Japan, Australia, Review, 2011 | To provide a description of principal aspects of policy and practice associated with orphan drugs and treatments of rare diseases, and give perspectives for 2011 on new and emerging approaches for addressing patient access. | Information has been combined from a wide variety of sources including the authors' knowledge with this topic. This is accompanied by relevant information from conferences, meetings and articles published from the last two decades and from policy documents released by applicable regulatory bodies. | Approximately 30 million in the EU and 25 million in the USA are affected by rare diseases. Annual orphan drug treatment costs may approach $500,000. The high price of orphan medicines together with scarce clinical data and difficulty in meeting standard cost‐effectiveness benchmarks raise significant issues for payers in allocating finite budgetary resources. Regulatory bodies have introduced a large amount of incentives to help sponsors develop and market orphan medicinal products. |
| Blankart (3) | Australia, Canada, England, France, Germany, Hungary, Netherlands, Poland, Slovakia ,Switzerland & The United States, Primary Research, 2011 | Use a set of indicators to identify differences, both in the availability of orphan drugs and in patient access to them, in 11 pharmaceutical markets. | A set of indicators were used to determine the availability and access in each country.  Data on availability was obtained from the relevant administrative or federal department such as the US FDA or Australian TGA. Data on patient access was obtained from the largest or most important payer in each country | Most orphan drugs had received marketing authorization in all countries, but the range of indications (HTA) differed by country. The broadest range was found in Australia. Authorization speed was the highest in the United States. The highest prices for orphan drugs were found in Germany and the United States, with the lowest in Canada, Australia and England. The prices of orphan drugs were high compared to non-orphan medicines, but most of the insurance plan provided coverage after a required payment threshold. |
| Boon (37) | The Netherlands, Health Reform Monitor, 2014 | To discuss the origins, governance and outcomes of a conditional reimbursement trajectory for orphan drugs in the Netherlands | The study utilised data regarding the history of the policy scheme from archives and websites of two cancer patient organisations, a medicinal specialist organisation, a federation representing pharmaceutical companies, the MOH and Parliament. The study also investigated the 12 orphan drugs that were listed under the orphan policy rule in the Netherlands and were investigated in by conducting interviews with key informants in the study period. | The Netherlands conditional reimbursement scheme is the result of years of debate and returning public pressure about imbalanced access to expensive drugs |
| Brabers  (18) | European Union, Primary Research, 2011 | To determine  whether the market exclusivity incentive of the EU  Orphan Drug Regulation results in a market monopoly  as or that absence of a follow-  on OMP for a rare disorder for which already an  approved OMP exists in the EU is a matter of time or  market size | The impact of market, product and disease related characteristics on follow-on OMP development in the EU was determined by comparing rare disorders with an approved OMP and no follow on OMP. It was then determined whether manufacturers continued development of a follow-on OMP upon approval of the first OMP for the rare disorder. For each rare disorder with an approved OMP, the EU register of orphan medicinal products was checked for follow on OMPs between April 2000 and 2010. | Follow-on OMP development was strongly associated with disease prevalence, first OMP turnover, disease class, age of disease onset and disease specific clinical or scientific output. Of 120 follow-on OMPs only one could be identified for which development was discontinued upon approval of the first OMP for the disorder. Additionally, sponsors of follow on OMPs often assumed their product have superior clinical efficacy compared to the first. |
| Cote (41) | United States, European Union, Commentary, 2012 | To try to identify the reasons for the main effects of orphan drug policies. The study also aims to highlight several implications of orphan drug policies of public health systems. | The study was a qualitative and inductive type. The empirical material consisted of a combination of objective/subjective and qualitative/quantitative data from three main sources: the remarks and comments of practitioners, statistical data, and factual and analytical elements from the literature. | The article provides a commentary regarding the US orphan drug act and two unforeseen effects of orphan drug policies; “they provide unique business opportunities for manufacturers” and “drugs approved through these policies are often accessible due to their high price”. |
| Denis (16) | Belgium ,France, Italy, The Netherlands ,Sweden & The UK, Comparative Study/ Review, 2010 | To compare regulatory aspects of rare disease and orphan drug markets in Belgium, France, Italy, the Netherlands, Sweden and the United Kingdom. | Information was derived from the international literature, analysis of legal texts, and a survey completed by national experts | Countries adopted different approaches in the regulation of orphan drugs and rare disease markets. This results in differences in the availability, pricing and reimbursement of orphan drugs vary between countries. Various pricing strategies exist to lower prices include public procurement in Sweden, profit controls in the United Kingdom, and price comparisons with other countries. Standards of evidence required for reimbursement differ between countries and can include: cost-effectiveness and/or budget impact of orphan drugs as well as societal considerations, such as whether the drug treats a life-threatening disease, are sometimes taken into account. |
| Denis (9) | European Union, Opinion, 2010 | To identify and discuss several issues surrounding orphan disease and drug policies in Europe. | Opinion piece utilising scientific literature | The current system of orphan designation allows for drugs for non-orphan diseases to be designated as orphan drugs. The study also found a low prevalence of a certain indication does always result in a low return on investment for the drug. High-quality evidence about the clinical added value of orphan drugs is rarely available at the time of marketing authorization, due to the low number of patients. The study recommends a balance between ethical and economic concerns. |
| Drummond (39) | United Kingdom, with reference to the wider EU, Commentary, 2007 | To discuss whether the standard methods of HTA are adequate for assisting decisions on patient access to and funding of orphan drugs and to outline a research agenda to help understand the societal value of orphan drugs and issues surrounding their development, funding, and use | Commentary piece accompanied by scientific literature | The study finds the current situation, where companies are given incentives to develop orphan drugs, yet, access to the drugs is limited by financial limitations, is ineffective for society, patients and the industry. In particular, incentives should go beyond market exclusivity to patient access and reimbursement. Standard HTA procedures may not fully capture the value of these medicines to society and therefore there are various inadequacies in the evaluation of orphan drugs. |
| Drummond (53) | European Union, Commentary, 2014 | The study aims to set out a roadmap whereby key policy problems relating to orphan medicines can be resolved. | A literature review was conducted in order to obtain an accurate view of the problems and to propose possible solutions | Current orphan drug policies are not fit for the purpose. Solutions should be implemented to: “clarify society’s views about the priority to be given to orphan drugs, revise the arrangements for pricing and reimbursement of orphan drugs, define the priorities for research into rare diseases and develop ‘joined up’ policies to deal with these issues.” |
| Dunoyer (49) | Global, Commentary, 2011 | To highlight areas in which novel approaches could facilitate regulatory approval and access to treatments for rare diseases. | Commentary piece accompanied by scientific literature | The study discusses several key ideas to accelerate access to orphan medicines. In particular; improving understanding of rare diseases, applying innovative strategies in clinical trials and the introduction of key regulatory and policy roles. |
| Dupont (48) | Belgium, Primary Researh, 2010 | To analyse the Belgian reimbursement decision of orphan drugs as compared to those of innovative drugs for more common but equally severe diseases, with special emphasis on the quality of clinical evidence. | The study used the Belgian National Insurance Agency administrative database and evaluated all submitted orphan drug files between 2002 and 2207. A quality analysis of the clinical evidence in the orphan reimbursement files was performed. The evaluation reports of the French “Haute Autorite de Sante”, including the five point parameter “Service Medical Rendu” (SMR) were examined to compare disease severity. Chi squared tests (at p<0.05 significant level) were used to compare the outcome of the reimbursement decisions between orphan and non-orphan innovative medicines. | Reimbursement and access to orphan medicines in Belgium depends upon assessment of their relative efficacy. This can delay access by a median of 65 days. Orphan drug status is a strong predictor of reimbursement. On average, lower levels of clinical and cost-effectiveness related evidence for granting reimbursement and providing access to therapy are accepted than for other “innovative” medicines. Post-marketing programmes/surveillance, with the aim of reducing the gap in clinical information and evidence is suggested. |
| Franco (1) | US, EU ,Japan, Australia ,Canada, Singapore & Taiwan, Review, 2013 | To describe and discuss the legal framework and the regulatory environment of orphan drugs worldwide | Scientific literature review | Orphan drug legislations and policies have been successful given that more medicines for rare diseases have been authorised since the implementation of these legislations and policies. |
| Garau (4) | France, Germany, Italy, Spain, Sweden, The Netherlands & The United Kingdom), Primary Research, 2009 | To compare the pricing and reimbursement (P & R) arrangements implemented in selected EU countries to make coverage decisions on orphan medicinal products (OMPs) and investigate whether these measures had an impact on OMP availability and access | Comparative Study of the selected EU Countries utilising data on coverage decisions on the first 43 OMP’s approved by the EMEA in its first eight years with data sources including an IMS database, health care body websites. The study also undertook a literature review and consultation of national experts. | Criteria informing P & R and coverage decisions vary substantially across the countries studied. The most common factors deemed important were the severity of the illness and lack of an adequate alternative treatment, a limited evidence base for approval and the high cost of OMP’s. |
| Hansen (45) | European Union, Review, 2012 | To give an overview from the available literature and experience of stakeholders, with a special focus on key strategies that made this segment a viable and ever growing market. In addition, it highlights the specificities and the common ground regarding Rare Diseases and Orphan Drugs compared to common diseases and classic drugs and suggests additional strategic options. | A literature review of relevant publications identified in the Medline database as well as on certain websites dedicated to Rare Diseases and Orphan Drugs was therefore used as the basis for this publication. This work was completed by interviews with stakeholders of the Rare Diseases and Orphan Drugs | Overview of the available literature and experience of stakeholders. It also highlights the specificities and the common ground regarding rare diseases and orphan drugs as compared to non-rare diseases and common drugs and suggests additional strategies and options in the field |
| Hughes-Wilson (55) | European Union, Review, 2012 | To derive a new assessment system for orphan drugs to give pricing and reimbursement decision-makers a tool to handle the different characteristics amongst new orphan drugs. | Literature Review and investigation into new systems for assessment for orphan drugs. | Analysis of criticism relating to the funding and reimbursement of orphan drugs. These criticisms are suggested in the turning of these ‘behaviours’ into criteria for a system to evaluate orphan drugs. A new assessment system is proposed based on specific evaluation criteria to enable member states to evaluate orphan drugs for pricing and reimbursement. |
| Iskrov (19) | Bulgaria (With reference to Eastern Europe), Primary Research, 2012 | Aims to present and analyse the results of a review on the access to Orphan Drugs in Bulgaria. It also gives a further analysis of how Eastern European Countries could deal with Orphan Drugs, combining EU level policies with their own national medicinal products legislation, organisational conditions of the health system and patient need and expectation. | Cross sectional observational study taking the total number of Orphan Drugs available on EU level, and filtering by the requirements and criteria of relevant Bulgarian legislation on OMP registration (designation), pricing and reimbursement to obtain the final number of available OMP in Bulgaria. The study also evaluates the average time period from market authorization to positive reimbursement decision by Bulgarian Health Authorities. | The article highlights several key aspects of orphan drug access in Bulgaria, including: a lack of epidemiological ad clinical data, difficult pricing procedures, limited public awareness, smaller market size as well as non-reimbursement of EU market authorized OMP’s. The study finds that 16 of the 61 currently registered in the EU, are available in Bulgaria. (Available by March 2011) |
| Iskrov (14) | EU,US, Australia, Japan, Review, 2014 | To explore the current rationale of post-marketing access to orphan drugs. | Scientific literature review including the development of an analytical framework for orphan drugs. | A critical analysis of Health Technology Assessment and reimbursement decision making decisions for orphan drugs. The article provides a description of general international themes regarding policies and regulation regarding HTA and reimbursement consisting of: cost-effectiveness criterion, lack of quality clinical evidence, impact of high prices on reimbursement decisions, budget impact analyses, ethical arguments and pricing/reimbursement as a whole. |
| Joppi (62) | European Union, Opinion, 2013 | To assess the methodological quality of Orphan Medicinal Product (OMP) dossiers and discuss possible reasons for the small number of products licensed. | Information about orphan drug designation, approval, refusal or withdrawal was obtained from the website of the European Medicines Agency and from the European Public Assessment Reports. | “From 2000 up to 2010, 80.9 % of the 845 candidate orphan drug designations received a positive opinion from the European Medicines Agency (EMA)’s Committee on Orphan Medicinal Products. Of the 108 OMP marketing authorizations applied for, 63 were granted. Randomised clinical trials were done for 38 OMPs and placebo was used as comparator for nearly half the licensed drugs. One third of the OMPs were tested in trials involving fewer than 100 patients and more than half in trials with 100–200 cases. The clinical trials lasted less than one year for 42.9 % of the approved orphan drugs.” |
| Kamusheva (20) | Bulgaria, Greece & Romania, Primary Research, 2013 | To make a cross-country comparison of the access to orphan medicines through reimbursement systems in three neighbouring Balkan EU member states: Bulgaria, Greece and Romania. | Comparative study of the orphan drugs included in the reimbursement drug lists (of authorized orphan medicines in the EU for May 2012 – October 2012). This information was obtained for orphan medicines with and without prior orphan designation from the list of orphan drugs in the EU, as well as a search of the officially published reimbursement lists of the three studied countries. A Literature search was conducted to identify related policy and legislative documents. The documents found were analysed according to the historical development, main stakeholders, rare diseases covered and the process of reimbursement approval. | The study found that the available and “accessible” OMP’s with EU authorization and prior orphan designation (of 69 OMP’s) in October 2012 was 24 (35.3%) in Bulgaria, 45 (66.18%) in Greece and 36 (52.94%) in Romania. Differences in pricing & reimbursement, purchasing power parity, financial and budget constraints were identified as root causes. |
| Kesselheim (54) | United States, Opinion, 2012 | To discuss ethical considerations in orphan drug approval and use | Opinion piece accompanied by scientific literature | Discussion of the orphan drug act, with specific focus on the lack of high-quality clinical evidence and safety data with regards to orphan drugs. The nature of the FDA to commonly waive strict criteria regarding clinical efficacy when assessing orphan drugs is discussed, with an attention to the ethical nature of these decisions. |
| Kockaya (38) | Turkey, Primary Research, 2014 | To “shed light on the use of orphan drugs in Turkey to aid further classifications of rare diseases and assessments of orphan drugs in the country”. | The study included orphan drugs recognised by the EU and published by the EMA. Sales information of the orphan drugs was extracted from IMS Turkey for 2008, 2009 and 2010. Sales information of non-authorized orphan products were extracted from the records of the TITCK. Published government prices for authorized and unauthorized drugs were extracted from the web-sites of the TITCK and the SGK. Orphan drug consumption depending on monetary value and volume was analysed using Microsoft Excel 2010. A detailed literature search of the PubMed Database was conducted. Eight relevant published articles were found, none of which related to the orphan medicinal market in Turkey. | The EMA has classified more than 60 orphan drugs, of which 50 entered the market in Turkey in the study timeline. The remaining 10 drugs are imported through an “early access procedure” for rare disease patients in need. The study finds that, while specific legislation and incentives to develop orphan drugs are currently not present in Turkey, recognised pricing and reimbursement policies are available, with all orphan drugs covered by reimbursement, regardless of market authorization status. Authorized OMPs are also all reimbursed without co-payments from patients. |
| Liu (23) | United States, EU, Japan, China, Review, 2010 | To analyse the strengths and weaknesses of the incentives included in orphan drug policies and regulations worldwide and describe the status of rare diseases in China. The study also aims to offer some recommendations for orphan drug legislation in China, based on local research on rare diseases. | Scientific literature review | Key barriers to patient access to orphan medicines include: high drug costs, low diagnose rates, low public awareness and differences in orphan medicine pricing policies |
| Llinares (61) | United States, European Union, Australia, Japan, Review, 2010 | To provide a regulatory overview about rare diseases | Landscape narrative review | The study Investigates, country by country, any relevant OMP legislation and relevant incentives. These include: orphan designation, marketing authorization, marketing exclusivity and financial incentives (tax deductions, fee waivers, grants/grant programmes, regulatory fee reductions). Other incentives include protocol assistance, priority review and exemptions from regulatory activities. |
| Logviss (43) | Latvia, EU, Review, 2014 | To determine situation in the field of rare diseases in Latvia and compare it with other European countries | (Narrative) literature review using the national plan for rare diseases, EUCERD reports, Orphanet data, Latvian and European regulations, publicly available data from the state agencies, and directly contacted drug manufacturers and wholesalers | “Currently 34 orphan drugs are available on Latvian market. Three medicines (8.8%) are included in the reimbursement drug list. 15 drugs (44.1%) were reimbursed within the framework of individual reimbursement system, and five drugs (14.7%) were provided within the program of medicinal treatment of rare diseases in children.” |
| Logviss (33) | Latvia, Primary Research, 2013 | “To determine orphan drugs associated with surgery (used pre, during or post-surgery) and their availability and access in Latvia” | The European Register of designated orphan products and EMA approved summary of product characteristics were analysed to find orphan drugs with approved labelled indications related to surgery. Drug availability and access in Latvia were determined by utilisation of data from the state agency of medicines of Latvia and the National Health Service. A literature review was also performed to compare the Latvian situation in the field of orphan medicines with that of other EU countries. | For the 15 orphan drugs identified, 6 are available in Latvia, and only one is included in the Latvian reimbursement list. The majority are oncology products. The Latvian reimbursement system focuses on therapeutic traditional cost-effectiveness. All other OMP’s are reimbursed through the individual reimbursement system of which a limit of 10,000 LVL (14,229 Euros) |
| Logviss (34) | Latvia, Primary Research, 2014 | To determine trends in reimbursement of orphan drugs in Latvia within the framework of the individual reimbursement system from 2008-2011. | Investigates the availability of two types of medicinal products intended for rare diseases in Europe. OMP’s with EU orphan designation and EU marketing authorization, and those with EU marketing authorization but without EU orphan designation (these products may have orphan designation internationally however). Availability of the OMP’s was determined through the use of the Latvian national register of Human Medicines. Data from the national health service (annual reports) was used to analyse individual reimbursement of each OMP. | Orphan drug reimbursement is the largest portion of drug reimbursement for individual patients in Latvia. The majority of orphan drugs are not included in Latvian reimbursement lists, due to a standard pharmo-economic evaluation which highlights therapeutic and cost effectiveness as the primary focus for drug inclusion. 3 of 29 OMP’s are available in Latvia, reimbursed. Orphan drugs can be individually reimbursed to a limit of 14,229 Euros. |
| Mariz (57) | European Union, Opinion, 2014 | To review and highlight specific regulatory mechanisms which are used or tailored to help in the development and licensing of products which receive an orphan designation | Opinion piece providing a review the post-designation incentives framework that exists in Europe under its Orphan Medicinal Designation System | The study finds that OMPs have obtained licenses under different regulatory mechanisms These have made these products available across Europe. Licenses for OMPs should make orphan drugs available in all member states. |
| Morel (60) | Italy, The Netherlands, United Kingdom, Sweden, Belgium, France, Germany, Primary Research,2013 | To examine the processes by which MEAs are implemented, identify, describe and classify MEAs applied to OMPs and analyse and compare identified MEAs related to OMPs within and between countries. | National Health Technology Assessments and Reimbursement Decisions on orphan drugs across seven EU countries were reviewed and main characteristics extracted. Identified MEAs were analysed and compared with attention to five themes: drug targets, geographical spread, type, declared rationale and evolution over time. | Confirmation of a variety of MEAs increasingly used by European payers to manage uncertainty associated with the introduction of orphan drugs. These uncertainties related to clinical, utilisation or budgetary concerns, |
| Pavlovic (22) | Serbia ,Bulgaria, Sweden, Primary Research, 2012 | To evaluate differences in access to orphan medicines between Serbia, Bulgaria and Sweden, | Comparative study involving a review of national reimbursement lists from each country crossed with the list of orphan drugs in Europe (EU authorized), published in July 2011. The analysis of regulatory traits was based upon a review of official documents and international literature regarding legislation around rare diseases and OMP’s. | Serbia had the lowest rate of orphan drugs available and reimbursed on the market at 16.3%, followed by Bulgaria (47.3%) and Sweden (56.6%). Differences in approaches for registration, pricing and reimbursement of orphan medicines, budget issues and purchasing power parity differences between countries were considered root causes. |
| Picavet (29) | European Union, Primary Research, 2012 | To evaluate orphan drug policies in Europe with key orphan drug experts and formulate informed policy recommendations regarding the future of European Drug policies and regulations. | Two pronged methodology, with a review of the international literature, and a two rounded Delphi Policy study with 47 European orphan drug experts from academia, with a regulatory background, patient organisation representatives and members of the pharmaceutical industry. The methodology of the study was carried out in tandem to evaluate Orphan Drug Policies in Europe in order to optimize patient access to Orphan Drugs. | The article focuses on drug regulations in Europe including: criteria for orphan designation, nature of designated products, institution in charge of orphan designation, assistance and guidance, marketing authorization and market exclusivity (procedure and length of market exclusivity), accelerated procedure, other incentives and compassionate use. The article discusses expert opinion on each of these factors, with a general positive consensus on most issues, with recommendations for change due to inter-country socio-economic inequalities influencing access to OMP’s. |
| Picavet (17) | Belgium, Primary Research, 2014 | To use a combination of qualitative research methods to examine which official and non-official factors influence reimbursement decisions for orphan drugs in Belgium | Six semi structured interviews with past or present members of the Drug Reimbursement Committee (DRG) were performed with a view to obtain an overview of the potential factors influencing reimbursement, Additionally, the existence factors were assessed in the reimbursement dossiers of all orphan drugs (64) for which an application for reimbursement was submitted between January 2002 and July 2013. The data was then analysed qualitatively by thematic analyses in order to analyse expert opinion and prevalence of various aspects present in reimbursement decision making, both official and non-official. | Considerations of expert opinion on official decision making factors including: therapeutic value, cost-effectiveness, budget impact and importance in clinical practice. Non official factors considered included: national price, price and reimbursement in other countries, patient organisations, expert opinion, quality of the branded drug versus the pharmaceutical compounding, media attention, innovative character, economic importance, ethical arguments and the political climate. |
| Picavet (30) | Belgium, The Netherlands, Czech Republic, France, Italy and the UK, Primary Research, 2014 | To investigate how drug and disease specific variables relate to orphan drug prices as well as to explore if certain country specific pricing and reimbursement policies affect the price level of orphan drugs. | Annual treatment costs per indication per patient were calculated for 59 orphan drugs with a publically available price in the countries studied. A multiple linear regression model was built with 14 drug and disease-specific variables. A Mann-Whitney U test was used to investigate whether a correlation between annual treatment costs of orphan drugs across countries with different pricing and reimbursement policies exists. | Significant associations found between the average annual treatment cost and seven variables among the countries studied. Lower annual treatment costs (prices) were associated with repurposed orphan drugs, orally administered orphan drugs or orphan drugs with an alternative treatment. Higher treatment costs were associated with orphan drugs with multiple orphan indications, chronic treatments and OMPs for which an improvement in overall survival or quality of life has been demonstrated. No association was found between annual treatment costs of orphan drugs (prices) across countries and different pricing and reimbursement regulation/policies across the studied countries. Differential pricing systems, in which price in dependent upon a country’s GDP, could enhance equal access to orphan drugs internationally. |
| Picavet (58) | European Union, Commentary, 2012 | To examine the arguments that argue in favour of granting special status to orphan drugs | Commentary accompanied by (scientific) literature | The study provides an examination of arguments in favour of granting special status to orphan drugs. The authors conclude that monetary arguments must carefully balance with social and ethical motives to avoid unfair treatment of patients with rare diseases. |
| Saikiran Reddy (28) | India, with reference to the United States, Australia, Japan and the EU, Review, 2014 | To put forward the challenges faced by rare disease drug development and the current scenario of orphan drug legislations in India | Narrative literature review | Discussion of the challenge faced by rare disease development and the current scenario of orphan drug legislation in India. The orphan drug legislation of the US, EU, Japan and Australia are discussed with the objective of informing similar legislation in India. |
| Reider (50) | United States, Commentary, 2000 | The article aims to provide a description of the orphan drug act, as well as its provisions: grants and protocol assistance, tax credits and market exclusivity. | Commentary accompanied by (scientific) literature | The article discusses accessibility considerations, such as the high prices of orphan drugs, co-payments/deductibles and monopolisation due to market exclusivity. |
| Rollet (46) | EU, Review, 2013 | To provide a complementary industry perspective on some of the key questions and misconceptions related to value-pricing and the business model for rare diseases therapies, with the aim of facilitating discussion and future progress in the field. | Narrative literature review | The article discusses four main ‘misconceptions’ regarding policies and pricing dynamics as well as rare disease business models which are commonly encountered. These include: the high price of OMP’s, supposed high budget impact of OMP’s, high return on investment for OMP’s, costs of manufacturing should determine prices and rare disease companies are making excessive financial returns as a result of OMP’s. |
| Rosenberg-Yunger (13) | Canada, Australia, Israel, Primary Research, 2011 | To describe the process of priority setting for two orphan drugs – Cerezyme and Fabrazyme – in Canada, Australia and Israel, in order to understand and improve the process based on stakeholder perspectives. | Qualitative case study analysis of how three independent drug advisory committees made decisions relating to the funding of Cerezyme and Fabrazyme Interviews were conducted with 22 informants, including committee members, patient groups and industry representatives.  The study involved in-depth qualitative interviews and collection of relevant documents and explored decision making in drug reimbursement of the two orphan drugs. | Orphan drug reimbursement decisions were based upon clinical evidence, cost-effectiveness and cost analyses. The study investigates the funding of the two drugs and the application of three main thematic rules for reimbursement including: “evidence”, the “rule of rescue” and “equity of access” |
| Schey (25) | Eurozone countries + UK. Primary Research, 2011 | To predict the total cost of orphan medicines in Europe between 2010 and 2020 as a percentage of total European pharmaceutical expenditure. | Disease-based epidemiological model created upon trends in the designation and approval of new orphan medicines, prevalence estimates for orphan diseases, historical price and sales data for orphan drugs in Europe. Two aspect analysis including: predicting the number of diseases for which new orphan drugs will be approved over 2010 – 2020 based on an EU registry of orphan medicines and estimating the average ex-factory drug cost across the orphan drug life cycle. | The budget impact of OMP’s has grown over the 10 years since the introduction of the EU orphan drug regulation in 2000. Driven primarily by the approval of new drugs for diseases in which no treatments were previously available. The budget impact has grown from 0% to 3.3% and is expected to plateau at around 4.6% by 2016. Drug costs are expected to fall in price by 25% from the 10^th^ year after the loss of intellectual property rights and marketing exclusivity of 10 years. |
| Seoane-Vasquez (42) | United States, Primary Research, 2008 | To analyse the characteristics of orphan drug development and approvals, and their sponsors, and to evaluate the effective patent and marketing exclusivity life of orphan NMEs approved in the US market during the 1983-2007 period. | Primary data sources were the FDA orange book, the FDA office of orphan drugs development and the US patent and trademark office. Data included all orphan designations an approvals listed by the FDA and all NMEs approved by the FDA during the study period. Data was updated through to December 31 2007 and summary descriptive statistics were computed for the variables used in the analyses with differences in proportions assessed using chi-square and fishers exact tests, with group comparisons utilising t-tests. SPSS 16 was used for the analysis. | 7- year market exclusivity had a modest impact on the overall orphan drug NME drug patent and market exclusivity life and 322 NDA (marketing) approvals for orphan indications. Discussion was included of US financial incentives for OMPs. Public programmes, federal regulations and polices overall support orphan drug research and development by companies in the United States. |
| Sharma (35) | United States, EU, Japan ,Australia, Canada ,India, Taiwan, Korea, Hong Kong, Singapore, Review, 2010 | To compare the policies and orphan drug incentives worldwide along with the challenges faced by the pharmaceutical companies | Narrative literature review | Comparison of policies and orphan drug incentives worldwide alongside the challenges faced by pharmaceutical companies. Recent developments are seen in orphan drug approval, the various drugs in the orphan drug pipeline and the future prospective for orphan drugs and diseases. |
| Simoens (51) | European Union, Review, 2011 | To conduct a review of the international scientific literature to provide insight into two policy aspects surrounding rare diseases and orphan drugs, i.e. the pricing and reimbursement of orphan drugs | Narrative literature review | The article provides an insight into the drivers of orphan drug pricing and reimbursement across the European Union due to: pricing of orphan drugs, monopolistic power of firms, price variations between countries (and domestic pricing, reimbursement and market structure that influence international price variations), costs of R&D and market access with regard to national policies, economic viability of orphan drugs, orphan biopharmaceuticals and reimbursement. |
| Simoens (56) | European Union, Opinion, 2012 | The article looks to comprehensively revisit each of the arguments used to attribute special market access status to orphan drugs for rare diseases by focusing on rarity of disease, severity and rule of rescue, incentives for orphan drug development, price of orphan drugs, efficacy and cost effectiveness of orphan drugs, and equity in access | Opinion piece accompanied by (scientific) literature | This article focuses on an argument regarding the revisiting of the special market access nature of orphan drugs with evidence supporting no societal preference for treating rare diseases, rather, *severe* diseases. |
| Song (40) | Japan, Opinion, 2013 | The article aims to provide an examination of the policies and regulatory approaches to orphan drugs in Japan | Opinion piece accompanied by (scientific) literature | Findings for policies and regulatory approaches to orphan drugs include :financial subsides for up to 50% of expenses for clinical and non-clinical research, 10 year marketing exclusivity, 15% tax credits on research costs, 14% reduction in corporate tax ,priority review, fast track approval, free protocol assistance and user fee waivers. In addition Japan implements pricing and reimbursement regulation by the Japanese National Health Insurance (NHI) system which negotiates prices with pharmaceutical companies. |
| Song (32) | United States, European Union, Australia, Japan, South Korea, Taiwan & China, Review, 2012 | To describe the current status of the regulation of rare diseases and orphan drugs in Asia and we comparatively analyse the regulation of rare diseases and orphan drugs worldwide in order to examine the challenges to and future perspectives on promoting research on rare diseases and development of orphan drugs in China and other Asian countries. | Narrative literature review | The article provides a discussion regarding the current status of the regulation of rare diseases and orphan drugs in Asia and provides a comparative analysis of the regulations around rare diseases and OMP’s with respect to the countries covered. The study also discusses the challenges to and future perspectives on promoting research on rare diseases and the development of orphan drugs in China and other Asian Countries |
| Stefanov (21) | Belarus, Bulgaria, Czech Republic,Hungary,Moldova,Poland,Romania,Russia,Solvakia,Ukraine, Review, 2009 | To review current rare diseases and orphan drug activities in Eastern European countries (EEC) | Narrative literature review | The article provides a conversation on rare disease and orphan drug activities in Eastern Europe including attention to: information and awareness, diagnosis and screening, clinical management and rehabilitation, patient associations and national plans on rare diseases. These factors are driven by differences in national policy and regulation regarding rare diseases and orphan medicines. Large disparities between both EEC countries and the wider EU are seen with regard to both ability to access relevant treatment and information. |
| Stolk (52) | WHO Members, Commentary, 2006 | To propose selection criteria for an Orphan Medicines Model List that could form a departure point for future work towards an extensive WHO Orphan Medicines Programme. | Commentary accompanied by (scientific) literature | In this article, the authors conclude that the WHO should include orphan drugs in its policy considerations by “composing a complementary Orphan Medicines Model List as an addition to the EML”. The authors state that this “complementary list of "rare essentials" could aid policy-makers and patients in, for example, emerging countries to improve access to these drugs and stimulate relevant policies.” |
| Stolk (24) | Austria, Denmark, Finland, Portugal, The Netherlands and Sweden, Primary Research, 2009 | To determine, for drugs used in an outpatient setting, how utilisation of centrally authorized drugs varies across a selection of EU member states. In particular the study was interested in determining whether drugs that have received an orphan medicinal status show a higher level of variability in use than centrally authorized medicines without an orphan status, and consequently are more vulnerable to heterogeneity in access and subsequent use. | Random selection of five orphan medicines and nine other drugs that were centrally authorized in the EU between January 2000 and November 2006. The utilisation of these drugs was compared in the countries studied, Utilisation data was expressed as defined daily doses per 1000 persons per year. Variability in use across countries was determined by calculating the relative standard deviation for the utilisation rates of individual drugs across countries. | No association between orphan medicine status and variability in use across countries. Drugs with an orphan status were more expensive and had higher “innovation score” than drugs without an orphan medicine status. These results indicate that this difference in access is not unique to drugs with an orphan status. |
| Tambuyzer (47) | United States, European Union, Opinion, 2010 | To provide an industry perspective on some of the common questions and misconceptions related to orphan drug development and its regulation, with the aim of facilitating future progress in the field. | Opinion piece accompanied by (scientific) literature | The article discusses policies and regulation of orphan drugs, including: public funding of orphan drug research and development, incentives (market exclusivity, orphan designation and marketing authorization) as well as regulation regarding drug pricing, access and reimbursement |
| Russell Teagarden (15) | United States, Opinion,2014 | This article aims to discuss the effect of the US orphan drug act and its effects on the availability of, and access to orphan drugs in the US | Opinion piece accompanied by (scientific) literature | The article provides an overview of the United States Orphan Drug Act, with particular reference to both the successes of the ODA; increased orphan drug designations and marketing approval as well as additional barriers to patient access. Issues include, primarily, the high per patient costs of orphan drugs. The article also provides suggestions on a co-ordinated systems approach to increase patient access to orphan drugs. |
| Thamer (36) | Canada, France, Japan, Sweden, The UK and the United States, Review, 1998 | This article aims to provide a cross-national comparison of orphan drug policies and its potential implications for the US orphan drug act. | Cross-national study examining the policies and regulations of other countries regarding orphan drugs. . Information was obtained from peer reviewed journals and from ‘grey literature’ | The article provides a Cross national comparison of the six countries studied, to compare public policies affecting the development and marketing of pharmaceuticals for rare diseases. The article provides a balanced analysis of the strengths and weaknesses of the US Orphan Drug Act, with reference to the policies and regulations of the other included countries |
| Tordrup (26) | England, Scotland, Sweden ,France, Germany ,Spain ,Italy ,Poland, Review, 2014 | This study aims to map out the policies currently in place in eight European countries regarding HTA and its application to the case of ODs and explore the implications these policies have for coverage decisions. | Formal processes within each country for the appraisal of ODs were identified and reviewed by searching the relevant government agency websites for “rare diseases” in the local language, supplemented by searches of the peer review literature | The article finds that although similarities exist in pricing and reimbursement decision making across countries, significant differences continue to exist which continue to result in differing access to orphan drugs across EU member states. These differences tended to be differences in pricing models and standards of evidence required for reimbursement decision making. |
| Trama (10) | Austria, Belgium ,Czech Republic, Denmark ,Estonia, Finland, Hungary, Ireland, Italy, Latvia, Slovakia and the UK, Primary Research, 2009 | To provide an overview of the availability of OMP’s in EU member states and to describe major challenges limiting the access to OMP’s. | Development and distribution of a questionnaire in collaboration with the European Organisation for Rare Diseases (EURODIS). The questionnaire aimed at assessing the OMP’s availability in the included EU countries. Twenty OMP’s authorized by the EMEA before October 2006 were included in the study, The questionnaire included: “ATC code”, “date of national market availability”, “possibility of a pre-marketing (accelerated) access programme in each country”, “distribution channel” and “reimbursement provided by insurance company or public agency and reimbursement rate (%)”. The questionnaire was distributed to either the Ministry of Health or national drug agencies of each country for the period (Nov 2002- 2006). | Market access and availability delays can be highly inconsistent across countries. In addition, the countries with the most OMP’s available to patients include Finland, France, Germany and Sweden, followed by Austria, Czech Republic, Denmark, Italy, Netherlands, Norway, Spain and the UK, with Iceland, Latvia and Lithuania being the worst off. |
| Villa (44) | Global, Commentary, 2009 | The articles aims to examine the possible reasons for the lack of adequate R&D investment in tropical diseases and proposes changes to orphan drug laws to make them more responsive to this issue | Commentary accompanied by (scientific) literature | The article finds that “the measures used in ODL may also be effective in boosting R&D for neglected tropical diseases, if appropriately adapted to this market. Second, small-sized companies, which have played a successful role in the development of orphan drugs for rare diseases, may also represent a good business strategy for the case of tropical diseases.” |
| Wellman- Labadie (31) | United States (EU, Japan), Primary Research, 2010 | The study investigates several issues associated with the United States Orphan Drug Act (ODA) 1983. | This study involved a comprehensive orphan drug database compiled from FDA data and corporate annual reports of major pharmaceutical companies. Analysis enabled the generation of a descriptive drug overview, as well as documentation of individual orphan drug lifecycles. The study analysed the constructed database using Systat 8.0 graphic and descriptive statistic tools (SPSS Inc, Chicago). The resulting trend analysis was presented utilising tables and figures constructed by Microsoft Office products. | 2002 products have obtained orphan drug designation, with 352 obtaining FDA approval. At least 9% of orphan drugs have reached ‘blockbuster status’ with profits over $200,000 million USD. 14 previously discontinued products have been ‘recycled’ as orphan drugs. 32% of Orphan Designations relate to cancer, High treatment costs, due to many orphan drugs obtaining “tier 4” medication status, lead to 20-33% co-payments for patients. |
| Westermark (59) | European Union, Outlook, 2011 | The article aims to provide a discussion regarding the outcomes of the first decade of EU orphan drug legislation with consideration of challenges faced and opportunities for orphan drug development and regulation into the next decade. | Outlook piece accompanied by scientific literature | The article “reflects on the outcomes and experience gained in the past decade, and contemplate issues for the future, such as catalysing drug development for the large number of rare diseases that still lack effective treatments.” |
| Zlatareva (27) | Bulgaria, Greece, Macedonia, Romania ,Serbia, Primary Research, 2013 | To Evaluate the access to (orphan) drugs for rare diseases and make a comparison with respect to the reimbursement systems (and of the drugs) in the countries studied. | Survey on the legislation and policies for rare diseases in the countries studied and on reimbursement coverage. Information on the authorized orphan drugs in Europe included the list of Orphan Drugs in the EU for the six month period (May 2012 to October 2012). This information was gathered from the official published drug reimbursement lists in each country and compared against EU lists. The Survey collected information on regulatory policies, the non-governmental sector, models of (patient) access and reimbursement strategies as well as statistical indicators and the geo-economic development of these countries. | Patients in Greece illustrated the best access to orphan drugs, followed by Romania and Bulgaria, with Serbia and Macedonia following. Economic development was found to limit health sector growth and access to orphan medicines. On average, the countries with well-constructed orphan drug plans, pricing and reimbursement strategies as well as higher GDP per capita, experienced greater access to OMPs. |
