# Supplementary material for: Access to Orphan Drugs: A Comprehensive Review of Legislations, Regulations and Policies in 35 Countries
Source: PLoS One. 2015 Oct 9;10(10):e0140002. doi: 10.1371/journal.pone.0140002 (PMC4599885; doi:10.1371/journal.pone.0140002)
Supplement: S4 Appendix — (DOCX) [file pone.0140002.s004.docx]

**Appendix Four: Studies excluded based upon inclusion and exclusion criteria after further review of full paper**

| **Author(s), Year of Publication / Article Title*, Type of Article*** | **Reason for exclusion** |
| --- | --- |
| A Hutchings, C Schey, R Dutton, F Achana & K Antonov. 2014 / Estimating the budget impact of orphan drugs in Sweden and France 2013–2020. *Original Research* | Non-Relevance |
| 1. Acosta, A., Ciapponi, A., Aaserud, M., Vietto, V., Austvoll-Dahlgren, A., Kösters, J P., Vacca, C., Machado, M., Ayala, D H D., Oxman. A D. 2014 / Pharmaceutical policies: effects of reference pricing, other pricing, and purchasing policies. *Review* | Non-Relevance |
| C McCabe, K Claxton, A Tsuchiya. 2005 / Orphan drugs and the NHS: should we value rarity? *Education/Debate* | Non-Relevance |
| D Krajnović, J Arsić, D Jocić, A M Georgiev, L Tasić, V Marinković. 2013 / Evaluation of Pharmacists Knowledge and attitudes regarding rare diseases and orphan drugs. *Original Research* | Non-Relevance |
| D Uguen, T Lönngren, Y Le Cam, S Garner, E Voisin, C Incerti, M Dunoyer, & M Slaoui. 2014 / Accelerating development, registration and access to medicines for rare diseases in the European Union through adaptive approaches: features and perspectives. *Letter to the Editor* | Non-Relevance |
| Davies, JE., Niedle,S., Taylor, DG. 2012 / Developing and paying for medicines for orphan indications in Oncology :utilitarian regulation vs equitable care*?, Mini-review* | Non-Relevance |
| Enzmann H, Lütz J. 2008. European incentives for orphan medicinal products*. Review* | Article in German |
| G Nisticò. 2011 / Orphan drugs assessment in the centralised procedure*. Review* | Non-Relevance |
| J T Matthews & L Glass. 2013 / The Effect of Market-Based Economic Factors on the Adoption of Orphan Drugs Across Multiple Countries. *Review* | Non-Relevance |
| Kreeftmeijer-Vegter AR, van Veldhuizen CK, de Vries PJ. 2012. Orphan drugs: availability, reliability and reimbursement. *Commentary* | Article in Dutch |
| P Kılıça, G Koçkayab, Ö Yems¸ena, C Tan, F H Öztuncaa , P Aksungura & S Kermana. 2013 / Orphan drug regulations in Turkey. *Communication piece* | Non-Relevance |
| Philip Wahlster, Shane Scahill, Sanjay Garg, Zaheer-Ud-Din Babar. 2014/ Identifying stakeholder opinion regarding access to “high-cost medicines”: A systematic review of the literature. *Review* | Non-Relevance |
| Picavet, E., Dooms, M., Cassiman, D., & Simoens, S. 2011 / Drugs for rare diseases: influence of orphan designation status on price. *Letter to the Editor* | Non-Relevance |
| Rappagliosi, A 2001 / Patient access to Orphan Drugs in the European Union. *Commentary* | Unable to retrieve full text article |
| Roll K, Stargardt T, Schreyögg J. 2011. Authorization and Reimbursement of Orphan Drugs in an International Comparison. *Original research* | Article in German |
| Soon, S.-S., Lopes, G., Lim, H.-Y., Wong-Rieger, D., Bahri, S., Hickinbotham, L., Jha, A., Ko, B.-S., MacDonell, D., Pwu, J.R.-F., Shih, R., Sirachainan, E., Suh, D.-C., Wale, J., Zhang, X., Wee, H.-L. 2014 / A call for action to improve access to care and treatment for patients with rare diseases in the Asia-Pacific region. *Letter to the Editor* | Non-Relevance |
| W Pinxten, Y Denier, M Dooms, JJ Cassiman, K Dierickx. 2012 / A fair share for the orphans: ethical guidelines for a fair distribution of resources within the bounds of the 10-year-old European Orphan Drug Regulation*. Paper* | Non-Relevance |

*Non-Relevance as a reason for exclusion refers to exclusion based on reasoning that the article did not describe any specific orphan drug legislation/regulation/policy in any countries
